# The zinc-finger protein ZC3H10 suppresses type I interferon responses during viral infection by repressing interferon-stimulated gene promoters

**DOI:** 10.1371/journal.pbio.3003881

**Published:** 2026-07-16

**Authors:** Xiaojing Dong, Xiaoyan Zuo, Xia Xiao, Shichao Ma, Lili Ren, Zhuo Zhou, Xiaobo Lei, Jianwei Wang

**Affiliations:** 1 NHC Key Laboratory of System Biology of Pathogens, State Key Laboratory of Respiratory Health and Multimorbidity, Christophe Merieux Laboratory, National Institute of Pathogen Biology, Chinese Academy of Medical Sciences & Peking Union Medical College, Beijing, P.R. China; 2 Key Laboratory of Pathogen Infection Prevention and Control, Ministry of Education, Beijing, P.R. China; 3 Beijing Key Laboratory of Surveillance, Early Warning and Pathogen Research on Emerging Infectious Diseases, Beijing Center for Disease Prevention and Control, Beijing, P.R. China; 4 Beijing Research Center for Respiratory Infectious Diseases, Beijing, P.R. China; 5 School of Public Health, Capital Medical University, Beijing, P.R. China; 6 State Key Laboratory of Common Mechanism Research for Major Diseases, Suzhou Institute of Systems Medicine, Chinese Academy of Medical Sciences & Peking Union Medical College, Suzhou, Jiangsu, P.R. China; Cleveland Clinic Florida, UNITED STATES OF AMERICA

## Abstract

Type I interferons (IFNs) play a central role in antiviral immunity by activating the JAK-STAT signaling pathway to induce interferon-stimulated genes (ISGs). Precise regulation of this response is critical to avoid pathological inflammation and autoimmunity, but the molecular mechanisms that restrain IFN signaling remain incompletely defined. Here, we performed a human whole-genome cDNA library screen and identified ZC3H10 as a negative regulator of the type I IFN response. Overexpression of ZC3H10 suppressed ISG expression following IFNβ stimulation and increased susceptibility to infection by Newcastle disease virus, human rhinovirus 16, Enterovirus A71, and SARS-CoV-2, whereas genetic deletion of ZC3H10 enhanced ISG expression and antiviral resistance. Mechanistically, ZC3H10 required nuclear localization, a functional nucleic acid-binding domain, and its coiled-coil domain to exert its inhibitory function. Furthermore, chromatin immunoprecipitation assays revealed that ZC3H10 directly binds to the TTTC motif within ISG promoters, thereby preventing their activation. Together, these findings establish ZC3H10 as a critical negative regulator of IFN signaling that functions to balance antiviral immunity.

## Abstract 2

Antiviral type I interferon responses must be tightly restrained to prevent damaging inflammation. This study identifies ZC3H10 as a novel negative regulator that binds TTTC motifs in interferon-stimulated gene promoters to limit antiviral gene expression during infection.

## Importance

While type I interferon (IFN) signaling is critical for antiviral defense, its overaction can drive pathological inflammation and autoimmunity, underscoring the need to understand the regulatory mechanisms that fine-tune this pathway. This study identifies ZC3H10 as a previously unrecognized negative regulator of type I IFN signaling. Distinct from known inhibitory factors that act upstream, ZC3H10 functions directly at the chromatin level by binding to the TTTC motif within interferon-stimulated gene (ISG) promoters, thereby limiting transcriptional activation. By elucidating both the molecular mechanism and the physiological relevance of ZC3H10-mediated repression, this work establishes ZC3H10 as a critical node in immune homeostasis. These findings position ZC3H10 as a potential target for enhancing antiviral immunity or mitigating IFN-driven inflammatory diseases.

## Introduction

Innate immunity serves as the host’s frontline defense against viral infections, deploying rapid, broad-spectrum protection through pattern recognition receptors (PRRs) [1,2], including retinoic acid-inducible gene I-like receptors (RLRs) [3,4], Toll-like receptors (TLRs) [5,6], NOD-like receptors (NLRs) [7,8], and the DNA sensor cyclic GMP-AMP synthase (cGAS) [9–11]. These receptors detect viral RNA, DNA, or other pathogen-associated molecular patterns (PAMPs), triggering signaling cascades that induce type I interferons (IFNs) [12,13]. Secreted IFNs bind the heterodimeric interferon alpha/beta receptor (IFNAR) composed of IFNAR1 and IFNAR2 subunits, activating the receptor-associated protein tyrosine kinases Janus kinase 1 (JAK1) and tyrosine kinase 2 (TYK2), which subsequently phosphorylate the transcription factors signal transducer and activator of transcription 1 (STAT1) and STAT2 [14, 15]. Phosphorylated STAT1 and STAT2 dimerize and associate with the interferon regulatory factor 9 (IRF9) to form the interferon-stimulated gene factor 3 (ISGF3) complex, which translocates to the nucleus and binds interferon-stimulated response elements (ISREs) in DNA, driving the transcription of interferon-stimulated genes (ISGs) [16]. ISG-encoded proteins exert multifaceted antiviral effects across the viral life cycle [17–20]. Recent studies classify ISGs into five functional clusters, with Cluster 3 enriched for antiviral effectors (e.g., ISG15, IFIT1, OAS, IFITMs), while others regulate RNA processing, metabolism, or inflammation [21].

Recent research has increasingly highlighted the multifaceted roles of IFNs, expanding their recognized functions beyond antiviral defense to include critical contributions to immune homeostasis and the pathogenesis of autoimmune diseases [22, 23]. Dysregulated type I IFN signaling has been implicated in several autoimmune disorders, such as systemic lupus erythematosus (SLE) [24], rheumatoid arthritis [25], and systemic sclerosis [26]. In these conditions, sustained IFN activation drives chronic inflammation and tissue damage, underscoring the necessity of precise regulation of IFN signaling pathways to maintain immune equilibrium and prevent pathological outcomes. Despite this importance, strategies to suppress excessive IFN responses remain understudied. Current approaches primarily focus on two key mechanisms: the downregulation of IFNAR expression at the cell surface [27, 28], which limits signal initiation, and the induction of negative regulators such as suppressor of cytokine signaling (SOCS) proteins [29–31], ubiquitin carboxy-terminal hydrolase 18 (USP18) [32, 33], and microRNA-mediated post-transcriptional control [34]. These mechanisms collectively highlight the intricate balance required to regulate interferon activity, offering potential therapeutic targets for diseases linked to aberrant interferon signaling.

The zinc finger protein family, renowned for its functional versatility, governs processes ranging from transcriptional regulation and protein ubiquitination to cell migration. Certain members, such as ZCCHC3, play critical roles in innate immunity: ZCCHC3 acts as a co-receptor for viral RNA, promoting TRIM25-mediated ubiquitination of retinoic acid-inducible gene I (RIG-I) and melanoma differentiation-associated protein 5 (MDA5) to amplify RLR signaling [35]. Meanwhile, CCCH-type zinc finger proteins, widely recognized for their RNA-binding capacity, regulate RNA metabolism [36]. Among these, ZC3H10 a CCCH-type RNA-binding protein harboring three essential zinc finger domains modulates RNA stability and processing. ZC3H10 has been implicated in mitochondrial function [37], microRNA splicing/maturation [38], and thermogenesis through binding the *UCP1* promoter to enhance brown adipose tissue activity [39]. Despite its diverse roles, the mechanism underlying its mitochondrial regulation remains unclear, and its potential involvement in immune processes has yet to be explored.

Through a genome-wide gain-of-function screen employing complementary DNA (cDNA) overexpression libraries, we identified ZC3H10 as a potent transcriptional suppressor of ISG activation. The molecular mechanism was further delineated through chromatin immunoprecipitation sequencing (ChIP-Seq), demonstrating ZC3H10’s direct binding to a 5′-TTTC-3′ tetranucleotide motif within promoters of ISGs. These findings position ZC3H10 as a critical regulator of interferon response homeostasis, suggesting novel therapeutic targets for modulating antiviral immunity.

## Results

### A systematic screen for regulators of the type I Interferon signaling pathway

**Fig 1:**
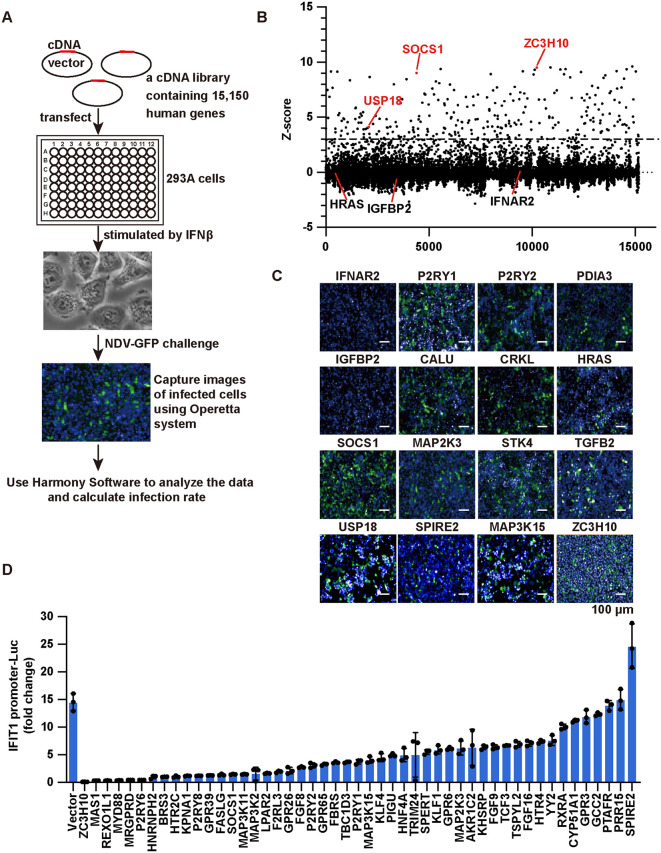
Genome-wide cDNA screening for antagonists of the type I interferon downstream signal pathway. **(A)** Schematic of the screening workflow. Plasmids containing the cDNA library (15,150 plasmids) were transfected individually into HEK293A cells. After 24 hours, IFNβ (400 U/mL) was added. Following 12 hours of stimulation, cells were infected with NDV-GFP at an MOI of 1. After an additional 16 hours, cells were fixed using 4% formaldehyde, stained with DAPI, and images were captured using the Operetta system to measure the NDV-GFP infection rate. **(B)** Primary screening results for each cDNA plasmid. Z-scores were calculated as (infection rate − average infection rate)/standard deviation of the infection rate. SOCS1, USP18 and ZC3H10 were highlighted in red. **(C)** Representative infection outcomes from the primary screening. Infectivity was indicated by GFP expression, and cell nuclei were stained with DAPI (blue). Scale bar, 100 μm. **(D)** Validation of candidate genes. The 50 top candidate genes from the primary screening were individually co-transfected with the IFIT1 promoter luciferase reporter. After a 24-hour incubation, IFNβ (400 U/mL) was added to the supernatant for stimulation. Twelve hours later, dual luciferase reporter gene expression was measured. IFIT1 promoter activity is shown as firefly fluorescence intensity normalized to Renilla fluorescence intensity. All data are presented as mean values ± SD. *p < 0.05, **p < 0.01, ***p < 0.001. The data underlying this Figure can be found in S1 Table, S1 Data, and S1 Raw Images.

To identify host factors that modulate the type I IFN signaling pathway, we conducted a cDNA library screen using HEK293A cells (Fig 1A). Cells were transfected with individual plasmids encoding 15,150 human genes. Twenty-four hours post-transfection, cells were stimulated with IFNβ for 12 hours to activate the IFN pathway, followed by infection with Newcastle disease virus (NDV) expressing green fluorescence protein (GFP) at an MOI of 1 for an additional 24 hours. NDV-GFP replication was calculated based on the proportion of GFP-positive cells via high-content screening, with the aim of identifying genes whose overexpression counteracts the antiviral effect of IFN. Using a Z-score threshold greater than 3, we identified 257 genes that counteracted IFNβ-mediated inhibition of NDV-GFP infection (Fig 1B and S1 Table). As expected, overexpression of IFNAR2, a positive regulator of IFN signaling, markedly reduced NDV-GFP infection, whereas SOCS1, a known negative regulator of IFN pathways, enhanced viral replication (Fig 1C).

To validate these findings, we performed a secondary screen using an IFIT1-promoter luciferase assay. From the 257 candidates, we selected the top 50 genes for further evaluation using the IFIT1-promoter luciferase reporter system. Each candidate plasmid was co‑transfected with the pFL‑IFIT1‑promoter‑Luc reporter and pRL‑SV40 (internal control). After 24 h, cells were treated with IFNβ for 12 h, after which luciferase activity was measured. The results showed that most of the top 50 genes inhibited IFIT1-promoter activation, and ZC3H10 demonstrated the most potent suppression of IFN-induced transcription, outperforming even SOCS1 (Fig 1D). Therefore, we selected ZC3H10 for further investigation.

### ZC3H10 overexpression inhibits the expression of ISGs

**Fig 2:**
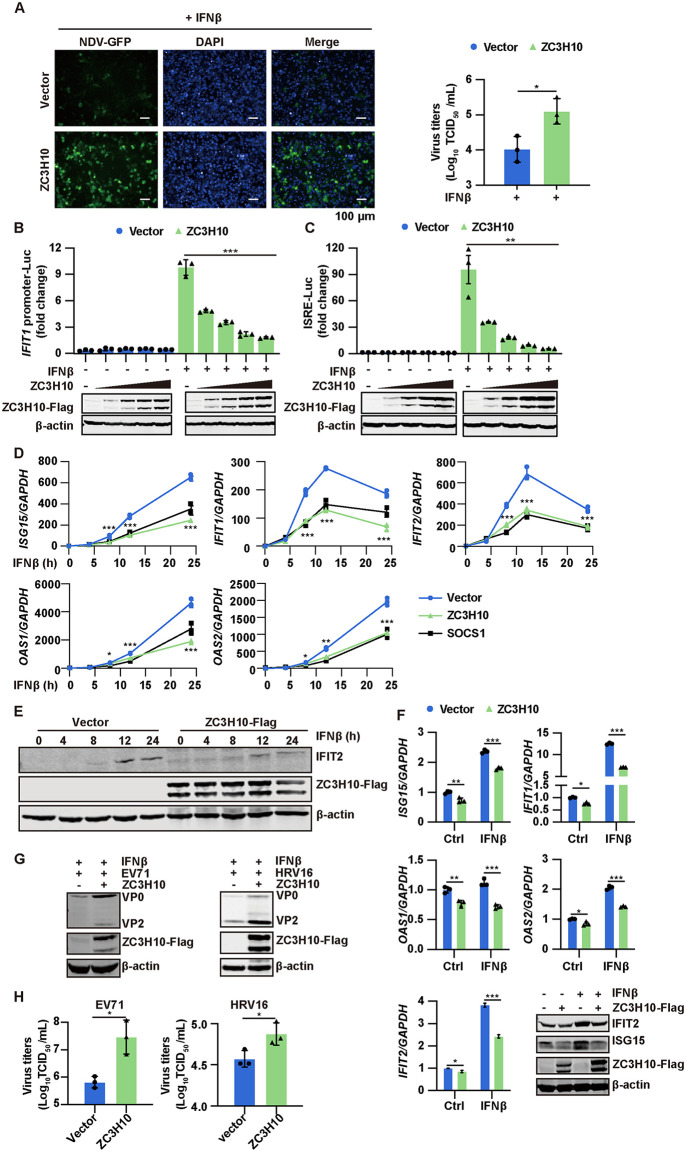
ZC3H10 inhibits ISG expression and promotes viral replication. **(A)** HEK293A cells were transfected with either empty or ZC3H10 plasmids. After 24 hours, cells were stimulated with IFNβ (400 U/mL). Twelve hours later, cells were infected with NDV-GFP. After an additional 16 hours, cells were fixed with 4% formaldehyde and DAPI was used to stain the nuclei. NDV-GFP is shown in green and nuclei in blue. Simultaneously, viral titers were measured by TCID_50_. Scale bar, 100 μm. **(B, C)** IFIT1 promoter luciferase (B) and ISRE luciferase (C) reporter plasmids were co-transfected into HEK293A cells with 0, 50, 100, 200, and 400 ng of ZC3H10 plasmids into HEK293A cells. After 24 hours, cells were either stimulated or not with IFNβ (400 U/mL). Twelve hours later, dual luciferase activity was measured, and ZC3H10 expression was assessed by western blot using anti-flag antibody. **(D)** HEK293A cells were transfected with 500 ng of empty plasmid (negative control), SOCS1 (positive control), and ZC3H10 plasmid. After 24 hours, IFNβ (400 U/mL) was added. At 0, 4, 8, 12, and 24 hours post-stimulation, mRNA expression of ISG15, IFIT1, IFIT2, OAS1, and OAS2 was measured by qRT-PCR. **(E)** HEK293A cells were transfected with 500 ng of empty plasmid (negative control) and ZC3H10 plasmid. After 24 hours, cells were stimulated with IFNβ (400 U/mL). At 0, 4, 8, 12, and 24 hours post-stimulation, protein expression of IFIT2 was detected. ZC3H10 expression was detected by using anti-Flag antibody. **(F)** RAW264.7 cells were transfected with 500 ng of empty plasmid (negative control) and ZC3H10 plasmid. After 24 hours, cells were either stimulated or not with IFNβ (100 U/mL). At 4 hours post-stimulation, mRNA expression of ISG15, IFIT1, IFIT2, OAS1, and OAS2 was detected by qRT-PCR, and protein expression of IFIT2 and ISG15 was assessed by western blot. ZC3H10 expression was detected by using anti-flag antibody. **(G–H)** HEK293A cells were transfected with 500 ng of empty plasmid or ZC3H10 plasmid. After 24 hours, IFNβ (400 U/mL) was added. Twelve hours post-stimulation, cells were infected with EV71 or HRV16 at an MOI of 0.5. Viral replication was assessed after another 16 hours by western blot **(G)**, and viral titers were measured by TCID₅₀ assay **(H)**. All experiments were repeated at least twice, with one representative result shown here. All data are presented as mean values ± SD. *p < 0.05, **p < 0.01, ***p < 0.001. The data underlying this Figure can be found in S1 Data and S1 Raw Images.

To further validate the functional role of ZC3H10 in IFN-mediated antiviral activity, we employed immunofluorescence and TCID_50_ analyses to assess the effect of ZC3H10 on NDV-GFP replication in the presence of IFN. These assays showed that ZC3H10 overexpression significantly antagonized IFNβ antiviral activity, enhancing NDV-GFP infection (Figs 2A and S1A). Given that IFNβ exerts its antiviral effects by inducing ISGs, we next examined the impact of ZC3H10 on ISG promoter activation. Luciferase reporter assays revealed that ZC3H10 suppresses both IFIT1 promoter- and ISRE-driven luciferase expression in a dose-dependent manner (Fig 2B and 2C). Consistent with these results, ZC3H10 also decreased the IFN-induced endogenous mRNA levels of several ISGs, including ISG15, IFIT1, IFIT2, OAS1, and OAS2 (Fig 2D). We further assessed IFIT2 protein expression levels by western blot, which showed that IFNβ induced IFIT2 expression in a time-dependent manner, whereas ZC3H10 overexpression decreased IFIT2 protein levels (Fig 2E). Importantly, this phenomenon was recapitulated in RAW264.7 macrophages, a well-established model for studying innate immune responses. In these cells, ZC3H10 downregulated mRNA levels of ISGs (ISG15, IFIT1/2, OAS1/2) in both the absence and presence of IFNβ stimulation (Fig 2F).

To further demonstrate the specificity of ZC3H10-mediated repression, we detected its effect on non-ISG genes, such as phosphoglucomutase 2 (PGM2) and euchromatic histone lysine methyltransferase 2 (EHMT2). The data showed that ZC3H10 overexpression did not alter the transcript levels of these two genes (S1B Fig). Another important remaining question was whether ZC3H10 modulates the expression of interferon regulatory factor (IRF)- and nuclear factor kappa B (NF-κB)-driven genes, which represent upstream transcriptional regulators of the antiviral response. Our findings demonstrated that ZC3H10 did not affect these key immune signaling axes (S1C and S1D Fig), indicating that its inhibitory function is specific to ISG transcription. Collectively, these results demonstrate that ZC3H10 specifically suppresses ISGs transcription.

To further validate the biological significance of these findings, we examined the effect of ZC3H10 on the IFN-sensitive viruses enterovirus 71 (EV71) and rhinovirus 16 (HRV16). Remarkably, ZC3H10 overexpression significantly enhanced the infectivity of both viruses, as measured by viral protein levels and viral titers (Fig 2G and 2H). Collectively, our results demonstrate that ZC3H10 functions as a potent negative regulator of the antiviral response by suppressing ISG expression, thereby supporting viral replication.

### Type I IFN signaling is upregulated in ZC3H10 knockout cells

**Fig 3:**
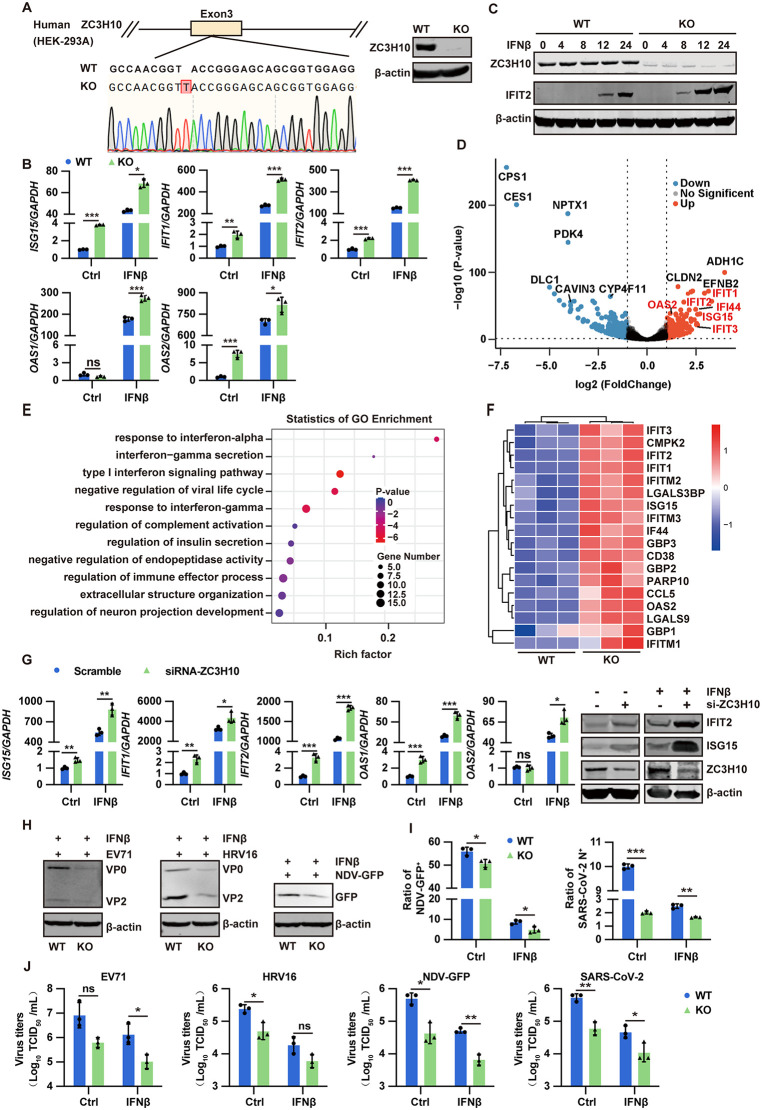
Enhanced ISG expression and reduced viral replication in ZC3H10 knockout cells. **(A)** HEK293A ZC3H10-knockout cells were generated by CRISPR-Cas9 genome editing. The left panel displays the sequences of the targeted region. The right panel shows ZC3H10 expression. **(B)** WT and KO cells were stimulated with or without IFNβ (400 U/mL). mRNA expression of ISG15, IFIT1, IFIT2, OAS1 and OAS2 was detected 12 hours post-stimulation by RT-PCR. **(C)** IFIT2 protein expression was detected by western blot at 0, 4, 8, 12, and 24 hours post-stimulation. ZC3H10 expression was detected by using anti-ZC3H10 antibody. **(D)** Volcano plot of differentially expressed genes (DEGs) comparing ZC3H10 knockout and wild-type cells. Each group consisted of three biological replicates. Red indicates upregulated DEGs, blue indicated downregulated DEGs. **(E)** Gene Ontology (GO) analysis of upregulated DEGs. **(F)** Heatmap of differentially expressed interferon-stimulated genes (DE-ISGs). **(G)** RAW264.7 cells were transfected with scramble siRNA and ZC3H10-siRNA. After 48 hours, cells were stimulated or not with IFNβ (100 U/mL). At 4 hours post-stimulation, mRNA expression of ISG15, IFIT1, IFIT2, OAS1, and OAS2 was detected by RT-PCR, and protein expression of IFIT2 and ISG15 was assessed by western blot. ZC3H10 was detected using anti-ZC3H10 antibody. **(H)** After stimulation with IFNβ (400 U/mL) for 12 hours, WT and KO cells were infected with EV71 (MOI = 0.5), HRV16 (MOI = 0.5), NDV-GFP (MOI = 1). Viral replication was assessed by western blot analysis after an additional 16 hours. **(I)** Cells seeded in 24-well plates were pre-treated with or without IFNβ (400 U/mL) for 12 hours, followed by infection with NDV-GFP. At 16 hours post-infection, cells were fixed with 4% paraformaldehyde and stained with DAPI. Images were then captured using an Operetta high-content imaging system. The percentage of infected cells were quantified by computing the ratio of virus protein-positive cells relative to the total number of nuclei (DAPI-stained). For SARS-CoV-2, cells were first transduced with a lentivirus expressing human ACE2. Cells were then stained using an antibody against the viral nucleocapsid (N) protein, followed by a 488-conjugated secondary antibody, and the percentage of N-positive cells were calculated using the same method. **(J)** Viral titers of EV71, HRV16, NDV, and SARS-CoV-2 in WT and ZC3H10 knockout (KO) cells were measured using a TCID₅₀ assay. All data are presented as mean values ± SD. *p < 0.05, **p < 0.01, ***p < 0.001. The data underlying this Figure can be found in S2–S3 Tables, S1 Data, and S1 Raw Images.

To further investigate the functional role of ZC3H10 in IFN responses, we generated a ZC3H10 knockout (KO) cell line using CRISPR-Cas9 technology. Successful disruption of the *ZC3H10* locus was confirmed by genome sequencing, and the absence of ZC3H10 protein expression was verified by western blot (Fig 3A). Having established these KO cells, we next examined whether ZC3H10 depletion affects the expression of ISGs. Quantitative real-time PCR (qRT-PCR) analysis revealed that ZC3H10 KO significantly upregulated the IFNβ-mediated expression of ISGs, including ISG15, IFIT1, IFIT2, OAS1, and OAS2 (Fig 3B). Notably, even in the absence of IFNβ stimulation, the basal expression levels of ISG15, IFIT1, IFIT2, OAS2 were also elevated in ZC3H10 knockout cells, suggesting that ZC3H10 exerts a repressive effect on ISG transcription under steady-state conditions. Consistent with the mRNA data, western blot analysis demonstrated that IFIT2 expression was markedly increased in ZC3H10 knockout cells relative to wild-type (WT) controls (Fig 3C).

To comprehensively investigate the transcriptional influence of ZC3H10, we performed RNA sequencing (RNA-Seq) comparing WT and ZC3H10 KO cells under basal conditions (without IFNβ stimulation). Differential expression analysis identified 259 upregulated and 241 downregulated genes in ZC3H10 KO cells compared to WT controls (Fig 3D and S2 Table). Gene Ontology (GO) analysis revealed that the upregulated genes were strikingly enriched in pathways associated with “type I interferon signaling” and “interferon-alpha response” (Fig 3E), indicating that ZC3H10 primarily represses genes involved in antiviral immunity. A heatmap depicting 18 differentially expressed ISGs further confirmed increased expression of IFITM family, GBP family, IFIT family, and ISG15 in ZC3H10 KO cells (Fig 3F), consistent with our qRT-PCR findings. To validate these observations in a physiologically relevant immune cell context, we performed siRNA-mediated knockdown of ZC3H10 in RAW264.7 macrophages. Similarly to the KO cell phenotype observed in HEK293A cells, ZC3H10 knockdown in RAW264.7 significantly enhanced ISG expression (Fig 3G), confirming that the repressive function of ZC3H10 is conserved across cell types.

Having established that ZC3H10 deficiency enhanced ISG expression, we next assessed the functional consequence for viral replication. Functional assays demonstrated that ZC3H10 KO cells exhibited markedly reduced replication of several IFN-sensitive viruses, as measured by viral protein expression levels, including EV71, HRV16, and NDV-GFP (Fig 3H). Furthermore, infections with NDV-GFP and severe acute respiratory syndrome coronavirus 2 (SARS-CoV-2) were substantially impaired in ZC3H10-deficient cells, and this effect was observed irrespective of the presence or absence of exogenous IFNβ (Fig 3I). Consistent results were obtained from viral titer assays (Fig 3J). Collectively, these results establish ZC3H10 as a critical negative regulator of the type I IFN response that promotes viral replication through broad suppression of ISG expression.

### The nuclear localization signal and zinc finger domains are crucial for ZC3H10-mediated ISG downregulation

**Fig 4:**
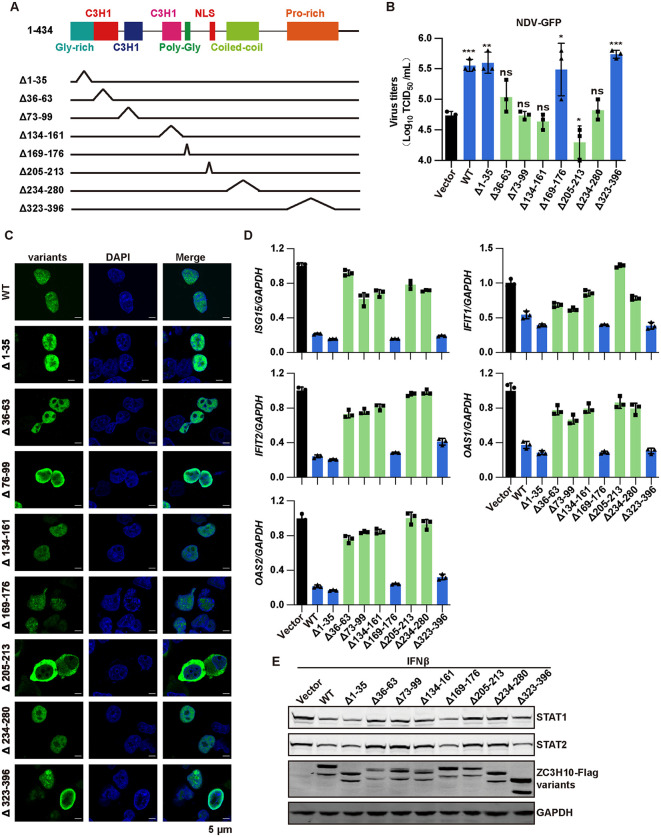
The nuclear localization signal (NLS) and zinc finger (ZF) domains of ZC3H10 are crucial for its function. **(A)** Schematic representation of ZC3H10 domains and its truncated variants. **(B)** HEK293A cells were transfected with either empty or plasmids encoding ZC3H10 variants. After 24 hours, cells were stimulated with IFNβ (400 U/mL). After an additional 12 hours, cells were infected with NDV-GFP at an MOI of 1. Sixteen hours later, viral titers were determined by TCID₅₀ assay. **(C)** HEK293A cells were transfected with plasmids encoding wild-type ZC3H10 or its truncated variants. After 24 hours, cells were fixed and stained with Flag antibodies. Green represents the variants and blue indicates nuclei. Scale bar, 5 μm. **(D, E)** HEK293A cells were transfected with either empty or plasmids encoding ZC3H10 variants. After 24 hours, cells were stimulated with IFNβ (400 U/mL). After an additional 12 hours, mRNA expression of ISG15, IFIT1, IFIT2, OAS1, and OAS2 (D) were measured by RT-PCR, as well as protein expression of STAT1 and STAT2 (E) were detected by western blot analysis. All experiments were repeated at least twice, with one representative result shown here. All data are presented as mean values ± SD. *p < 0.05, **p < 0.01, ***p < 0.001. The data underlying this Figure can be found in S1 Data and S1 Raw Images.

ZC3H10 is a CCCH-type zinc finger protein known to bind RNA and contribute to microRNA maturation [38], as well as to regulate transcription by interacting with the distal promoter of *UCP-1* [39]. Structurally, ZC3H10 contains three zinc finger domains (ZF1–ZF3), a nuclear localization signal (NLS), a coiled-coil region, an N-terminal glycine-rich region, and a C-terminal proline-rich region (Fig 4A). To identify the domains required for ZC3H10-mediated suppression of type I IFN signaling, we generated a series of truncation mutants and assessed their effects on NDV-GFP infection (Fig 4A). Overexpression of wild-type ZC3H10 significantly enhanced viral replication as expected. However, deletion of the NLS (ΔNLS, 205–213 aa), any of the three ZF domains (ΔZF1: 35–63 aa; ΔZF2: 73–99 aa; ΔZF3: 134–161 aa), or the coiled-coil domain (Δcoiled-coil, 234–280) abolished this enhancing effect, indicating that nuclear localization, intact zinc finger motifs and coiled-coil domain are critical for ZC3H10 function (Fig 4B). Subcellular localization studies confirmed that only the ΔNLS mutant was retained exclusively in the cytoplasm, whereas wild-type ZC3H10 and all other mutants localized to the nucleus (Fig 4C). These findings suggest that the NLS is necessary for nuclear import, while the zinc finger and coiled-coil domains, despite not affecting subcellular distribution, are indispensable for downstream functional activity.

Consistent with the viral replication data in Fig 4B, mutants lacking the NLS, any ZF domain, or coiled-coil domain lost the ability to suppress transcription of ISGs as assessed by qRT-PCR (Fig 4D). Furthermore, in contrast to wild-type ZC3H10, which suppressed IFNβ-induced expression of STAT1 and STAT2, the ΔNLS, ZF, and coiled-coil domain deletion mutants failed to reduce STAT1 and STAT2 protein levels (Fig 4E). Collectively, these results demonstrate that the NLS, the three ZF domains, and coiled-coil domain are essential for ZC3H10 to inhibit the IFN signaling pathway and promote viral replication.

**Fig 5:**
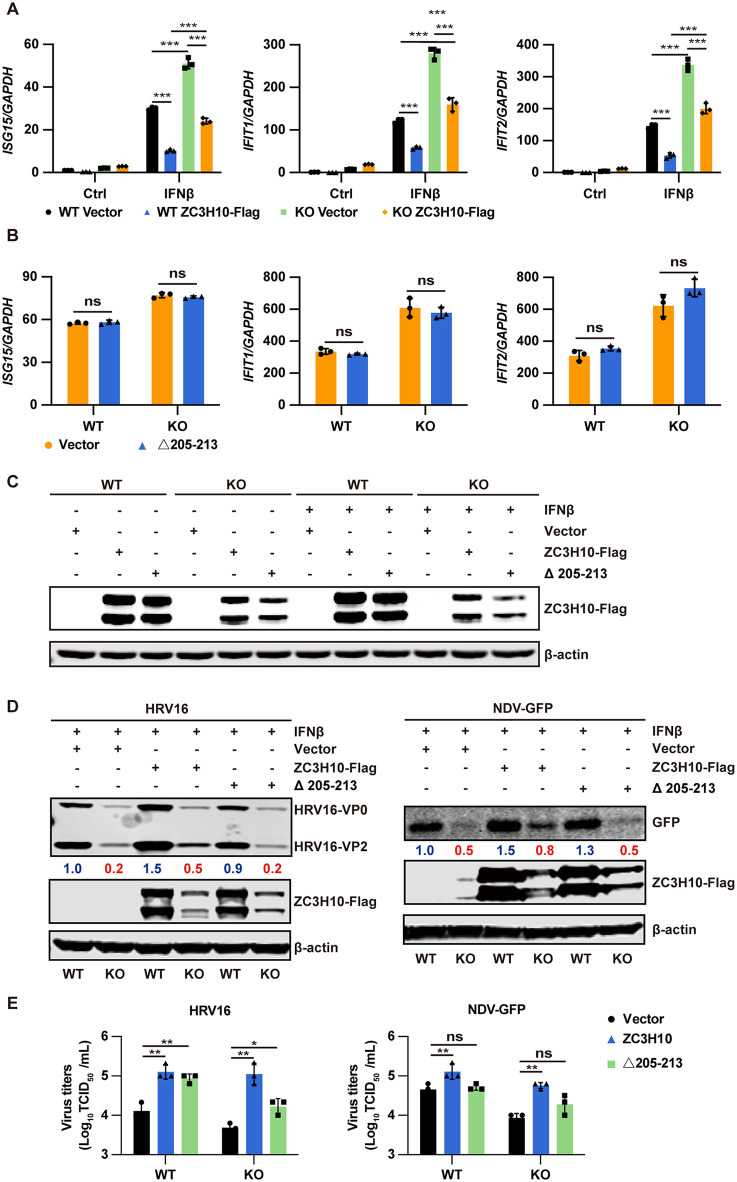
Re-expression of ZC3H10 in ZC3H10-deficient cells restores its inhibitory effect on ISG expression. **(A)** WT and KO cells were transfected with empty vector or ZC3H10 plasmid. After 24 hours, IFNβ (400 U/mL) or control medium was added. Following 12 hours of stimulation, mRNA expression of ISG15, IFIT1, and IFIT2 was measured by RT-PCR. **(B)** WT and KO cells were transfected with empty vector or NLS deletion mutant. Then, the cells were treated as described in panel **A. (C)** The expression of ZC3H10 and NLS deletion mutant was detected by western blot. **(D, E)** WT and KO cells were transfected with empty vector, ZC3H10 plasmid or NLS deletion mutant. After 24 hours, IFNβ (400 U/mL) was added. Following 12 hours of stimulation, cells were infected with HRV16 (MOI = 0.5) or NDV-GFP (MOI = 1). After another 16 hours, viral replication was assessed by western blot analysis **(D)**, and viral titers were measured by TCID_50_ assay **(E)**. Grayscale intensity of viral protein was quantified using Odyssey Image Studio software. All experiments were repeated at least twice, with one representative result shown here. All data are presented as mean values ± SD. *p < 0.05, **p < 0.01, ***p < 0.001. The data underlying this figure can be found in S1 Data and S1 Raw Images.

To further validate the role of ZC3H10, we performed reconstitution experiments. Reintroduction of wild-type ZC3H10 suppressed ISG expression in WT cells, consistent with the above results. As expected, expression of ZC3H10 in KO cells restored its inhibitory effect on ISG expression to levels comparable to those in WT cells (Fig 5A and 5C). However, reintroduction of the NLS-deficient mutant failed to inhibit ISG expression in either WT or KO cells (Fig 5B and 5C). Consistently, in viral replication assays, ZC3H10 knockout reduced HRV16 and NDV-GFP viral protein expression, and reintroduction of wild-type ZC3H10 partially restored viral protein expression in the knockout background (Fig 5D). Viral titer assays yielded similar results (Fig 5E). Together, our data suggest that the nuclear localization, ZF domains and coiled-coil domain of ZC3H10 contribute to its regulatory function, likely via DNA interactions, enabling suppression of IFNβ signaling.

### ZC3H10 inhibits ISG expression by binding to the TTTC motif of ISG promoters

**Fig 6:**
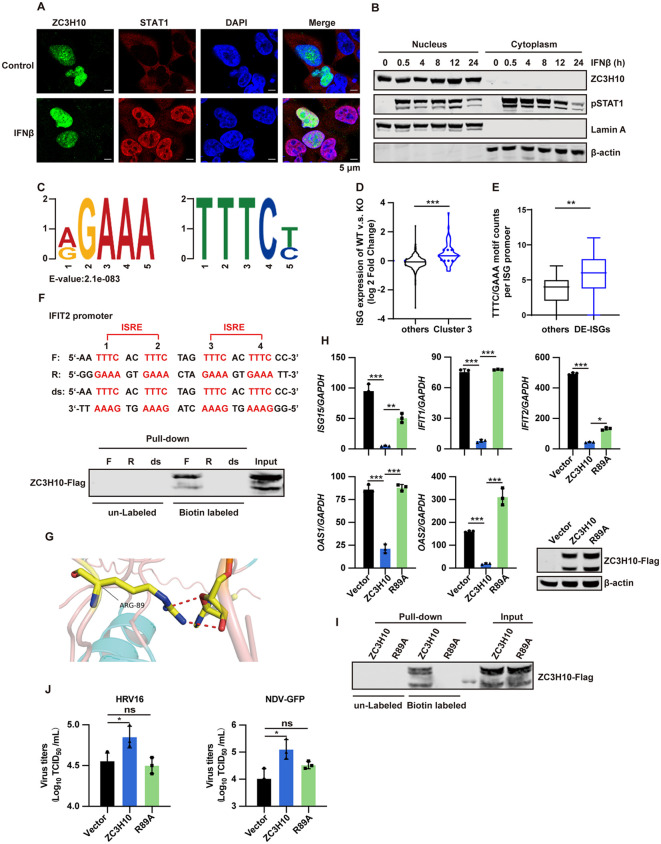
ZC3H10 can bind to the TTTC motif within the promoters of ISGs. **(A)** A549 cells were transfected with ZC3H10 plasmid and stimulated with or without IFNβ. After 6 hours, immunofluorescence was used to detect the expression of ZC3H10 (green) and STAT1 (red). Scale bar, 5 μm. **(B)** Following stimulation with IFNβ for the indicated time periods, HEK293A cells were harvested. Nuclear and cytoplasmic fractions were separated using a nuclear-cytoplasmic extraction kit. Protein expression of ZC3H10 and phosphorylated STAT1 was analyzed by western blot using anti-ZC3H10, and anti-p-STAT1 antibodies. Lamin A served as a nuclear loading control, and β-actin served as a cytoplasmic loading control. **(C)** Enriched motifs from ChIP-Seq analysis. **(D)** Gene expression of cluster 3 ISGs compared with other ISGs from RNA-Seq results in Fig 3D. **(E)** The number of TTTC/GAAA motifs in the promoter regions of DE-ISGs compared with other ISGs from RNA-Seq results in Fig 3D. **(F)** HEK293T cells were transfected with ZC3H10. Nuclear extracts were obtained by nuclear-cytoplasmic separation. Double-stranded DNA and sense/antisense single-stranded IFIT1 promoter oligos were used to pull down ZC3H10 protein. Five percent of the input and DNA-bound ZC3H10 protein were analyzed by western blot. **(G)** The binding sites of ZC3H10 protein and the ISRE sequence were predicted by AlphaFold3. **(H)** HEK293A cells were transfected with empty vector, ZC3H10, or R89A mutant plasmid. After 12 hours of stimulation with IFNβ, mRNA expression of ISG15, IFIT1, IFIT2, OAS1 and OAS2 was measured. ZC3H10 and R89A proteins were analyzed by western blot. **(I)** Sense single-stranded DNA oligos of the IFIT1 promoter were used to pull down ZC3H10 and R89A mutant proteins. Five percent of the input and DNA-bound ZC3H10 proteins were analyzed by western blot. **(J)** HEK293A cells were transfected with plasmids encoding wild-type ZC3H10 or R89A, with an empty vector serving as a control. At 24 h post-transfection, cells were stimulated with IFNβ (400 U/mL) for 12 hours. Then cells were infected with HRV16 (MOI = 0.5) or NDV-GFP (MOI = 1). At 16 hours post-infection, total cultures were harvested. After freeze-thaw cycles, viral titers were determined by TCID_50_ assay. All experiments were repeated at least twice, with one representative result shown here. All data are presented as mean values ± SD. *p < 0.05, **p < 0.01, ***p < 0.001. The data underlying this figure can be found in S1 Data and S1 Raw Images.

To investigate the mechanisms by which ZC3H10 inhibits ISG expression, we first examined whether its expression and localization are influenced by interferon stimulation. Cells were treated with IFNβ, and immunofluorescence analysis revealed that ZC3H10 remained constitutively nuclear, with no discernible changes in expression or nuclear localization following IFNβ treatment (Fig 6A and 6B). These observations suggest that ZC3H10 functions independently of IFN-induced signaling events in the cytoplasm. Consistent with this notion, we found that overexpression of ZC3H10 did not impair STAT1 nuclear translocation upon IFNβ stimulation (Fig 6A).

We next assessed whether ZC3H10 negatively regulates IFNβ signaling at the receptor level. qRT-PCR analysis revealed that ZC3H10 overexpression did not alter the transcriptional expression levels of IFNAR1/IFNAR2 (S2A Fig). Given that ZC3H10 did not affect upstream IFNβ signaling components, we hypothesized that ZC3H10 directly regulates ISG transcription. To test this hypothesis, we examined whether ZC3H10 influences ISG mRNA stability. HEK293A cells overexpressing ZC3H10 were treated with actinomycin D to block transcription, and the decay rates of representative ISG transcripts were monitored. As shown in S2B Fig, ZC3H10 did not significantly alter the degradation rates of ISG mRNAs.

We therefore investigated whether ZC3H10 directly associates with ISG gene promoters to regulate transcription. To this end, we performed ChIP-Seq using a flag-ZC3H10 overexpression system. Chromatin fragments were immunoprecipitated with an anti-flag antibody, and the enriched DNA was subjected to high-throughput sequencing. Motif enrichment analysis showed that ZC3H10 preferentially bound the 5′-TTTC-3′ sequence and its complementary 5′-GAAA-3′ motif (Fig 6C). Notably, both of which reside within the canonical ISRE sequence (5′-A/GGTTTCN(1-2)TTTCC/T-3′), a well-characterized regulatory element present in the promoters of numerous ISGs. Previous studies have categorized ISGs into five clusters based on their expression patterns, with cluster 3 predominantly featuring antiviral effectors such as the OAS and IFIT families, alongside critical regulators including USP18, STAT1, and IRF9 [22]. This cluster showed strong enrichment for the ISRE motif, which is recognized by the interferon-stimulated gene factor 3 (ISGF3) complex. Our RNA-Seq analysis demonstrated that the expression of cluster 3 genes was markedly increased in ZC3H10 knockout cells compared to WT controls (Fig 6D). Within this cluster, we identified 18 ISGs with significantly elevated expression levels in ZC3H10-deficient cells, which we designated as differentially expressed ISGs (DE-ISGs). To assess whether DE-ISGs are enriched for ZC3H10 binding sites, we quantified the presence of the TTTC and GAAA motifs in their promoter regions (Figs 6E and S3A). Notably, we found that DE-ISGs harbored significantly more of these motifs compared to other ISGs (Fig 6E). This finding supports the model that ZC3H10 suppresses ISG expression by directly binding to these motifs within their promoter regions.

To further validate the ChIP-seq results demonstrating ZC3H10 binding to the TTTC motif, we performed a DNA pull-down assay using biotinylated oligonucleotides. Nuclear extracts containing Flag-ZC3H10 were incubated with DNA oligonucleotides containing the wild-type TTTC motif within the ISRE sequence of the *IFIT2* promoter. This assay revealed that ZC3H10 specifically interacted with oligonucleotides encompassing the wild-type ISRE sequence (Fig 6F). As a control, we synthesized oligonucleotides harboring a mutant ISRE sequence and found that the binding of ZC3H10 to the mutant ISRE was markedly diminished (S3B Fig). These results demonstrate that ZC3H10 preferentially binds the ssDNA form of the ISRE containing the TTTC motif.

Previous studies have demonstrated that ZC3H10 requires phosphorylation at serine 126 (S126) to bind the distal promoter region of *UCP1*, thereby activating its transcription and promoting thermogenesis [39]. To determine whether this phosphorylation event is also required for ZC3H10-mediated repression of ISGs, we assessed the functional consequences of S126A and S126D phosphor-mutants. Notably, neither mutation affected the ability of ZC3H10 to repress ISG expression (S3C Fig), indicating that S126 phosphorylation is dispensable for this function.

To gain structural insight into the molecular basis of ZC3H10-mediated ISG repression, we employed AlphaFold3 to model the binding of ZC3H10 to the ISRE sequence (5′-AGTTTC-3′). The predicted model identified arginine 89 (R89) as a critical residue for DNA binding (Fig 6G). To experimentally validate this prediction, we generated a ZC3H10 mutant in which arginine 89 was substituted with alanine (ZC3H10-R89A). Overexpression of the R89A mutant in HEK293A cells failed to suppress ISG expression, in contrast to wild-type ZC3H10 (Fig 6H). Furthermore, DNA pull-down assays demonstrated that the R89A mutant exhibited severely impaired binding to the ISRE-containing oligonucleotide (Fig 6I). Consistent with this functional requirement, the R89A mutant also lost the ability to antagonize type I interferon signaling and promote viral replication (Fig 6J), further underscoring the physiological relevance of this residue. Collectively, these results demonstrate that ZC3H10 binds directly to ISRE sequences through a mechanism dependent on Arg89 and that this DNA-binding activity is essential for its inhibitory function in type I IFN signaling.

## Discussion

Interferons play a central role in antiviral defense by triggering the expression of IFN-stimulated genes through the JAK-STAT signaling pathway. While essential for immune responses, their dysregulation fuels chronic inflammation and autoimmune disorders. In this study, we conducted a host DNA overexpression screen to identify host factors that modulate the type I IFN signaling pathway. The identification of known negative regulators, including SOCS1 and USP18, provides convincing validation for our screening approach. The recovery of these known repressors confirms that our experimental design—using IFNβ priming followed by NDV-GFP infection—effectively captured biologically meaningful modulators of the antiviral state. Beyond these validating hits, our screen also uncovered several unreported hits, such as ZC3H10, P2RY1, P2RY2, STK4, LPAR2 and MAP3K15, suggesting that the regulatory network governing IFN signaling is far more diverse and interconnected than previously appreciated. While the present study focused on ZC3H10 as a representative candidate, these additional hits may also function as previously unrecognized suppressors of the IFN pathway and warrant further investigation in future studies.

Among these candidates, we focused on ZC3H10 and identified it as a novel, potent negative regulator of the type I IFN pathway. Functionally, ZC3H10 acts as a specific transcriptional repressor of ISG, thereby promoting the replication of IFN-sensitive viruses. Mechanistically, ZC3H10 directly binds to a TTTC motif within the ISRE of ISG promoters in a manner dependent on its nuclear localization signal, zinc finger domains, coiled-coil domain and a critical arginine residue. Together, these findings establish ZC3H10 as a key node in the fine-tuning of antiviral immunity, with implications for both viral pathogenesis and the control of inflammatory responses.

ZC3H10 suppresses ISG transcription via a mechanism distinct from the classical negative regulators, SOCS1 and USP18. SOCS1 inhibits IFN signaling by targeting JAK tyrosine kinase activity, thereby suppressing STAT1 phosphorylation and downstream gene activation [40, 41]. USP18 acts in a STAT2-dependent manner, recruiting to IFNAR2 and preventing the ternary complex formation necessary for IFN receptor signaling [42]. It also negatively regulates NF-κB signaling, further modulating inflammatory responses [43–45]. In contrast, ZC3H10 directly binds to TTTC/GAAA-rich sequences within ISG promoters to suppress their transcription. Notably, ZC3H10 does not affect NF-κB-driven genes or IRF activation, demonstrating its specificity for the ISG repertoire. However, the precise molecular mechanism by which ZC3H10 represses transcription once bound to DNA remains unknown. Our results indicate that ZC3H10 does not compete with ISGF3 for binding to ISRE elements. One possibility is that ZC3H10 recruits chromatin-modifying enzymes, such as histone deacetylases (HDACs) or methyltransferases, to promote a repressive chromatin state at ISG promoters. Future studies will be required to test this hypothesis and to determine how ZC3H10-mediated repression is relieved during viral infection or IFN signaling.

Our transcriptomic and chromatin-binding analysis collectively indicates that ZC3H10 represses a subset of ISGs by directly binding to TTTC/GAAA-enriched promoter regions, with arginine 89 playing a critical role in this interaction. This mechanism bears analogy to APOBEC3A, which recognizes TTTC motifs within the HIV-1 LTR to inhibit transcription and similarly targets TTTC motifs in the ISRE of the ISG15 promoter to modulate ISG activation [46]. These parallels suggest that ZC3H10 may function as part of a broader regulatory network in which sequence-specific DNA-binding proteins repress innate immune genes by targeting TTTC-rich elements. Therefore, our findings position ZC3H10 as a previously unrecognized transcriptional repressor of ISGs and point to TTTC motif recognition as a conserved mechanism for controlling antiviral gene expression.

The mechanism by which ZC3H10 binds single-stranded DNA in vitro can be considered in the context of transcription initiation. During this process, RNA polymerase II and associated transcription factors induce transient strand separation, generating single-stranded DNA “bubbles” at promoter regions. Canonical ssDNA-binding proteins such as replication protein A (RPA) are recruited to exposed ssDNA during DNA metabolism, transcription-induced DNA melting at promoter regions generates transient ssDNA structures that can serve as platforms for regulatory protein binding [47]. We hypothesize that ZC3H10 recognizes and binds to these transient ssDNA regions at specific promoters, thereby gaining access to chromatin. This binding may prevent transcription initiation or act as a physical barrier that blocks RNA polymerase II elongation. Additionally, we acknowledge certain limitations of our ChIP-Seq approach using a Flag antibody. First, overexpression creates a non‑physiological condition that may promote non‑specific chromatin binding. Second, the appended protein tag could perturb the structural folding of ZC3H10, altering its native conformation and consequently affecting protein function. A native ZC3H10 antibody, if available, would offer a more direct and physiologically relevant approach for future validation.

One intriguing aspect of our study is that ZC3H10 expression and subcellular localization remain unchanged upon IFN stimulation (Fig 6A and 6B), suggesting it is not an inducible component of the IFN signaling cascade. Unlike classical negative feedback regulators such as SOCS1, which are transcriptionally upregulated following pathway activation, ZC3H10 appears to function as a constitutively expressed surveillance factor or checkpoint protein. This constitutive expression positions ZC3H10 to temper ISG activation under both basal and stimulated conditions. Several key questions remain for future investigation. First, it will be important to determine whether ZC3H10 acts in vivo as a steady-state gatekeeper or an inducible shut-off mechanism. However, our initial attempts to address this question were hampered by the embryonic lethality of constitutive ZC3H10 knockout mice. Although we obtained heterozygous ZC3H10 mice, protein expression of ZC3H10 was not significantly reduced compared to wild-type controls. Unfortunately, this led us to not observe a significant phenotype in vivo. These results suggest that ZC3H10 may be essential for development. Moving forward, conditional knockout approaches, particularly lung-specific deletion, may offer a viable alternative to circumvent embryonic lethality and enable tissue-restricted functional studies. Second, investigating potential cooperation between ZC3H10 and established negative regulators such as SOCS and USP18 will be essential. These proteins operate at distinct levels of the pathway, SOCS1 targets JAK kinase activity, USP18 interferes with receptor complex formation, and ZC3H10 acts directly at ISG promoters. Whether they function independently, redundantly or synergistically to fine-tune innate immunity remains an open question.

**Fig 7:**
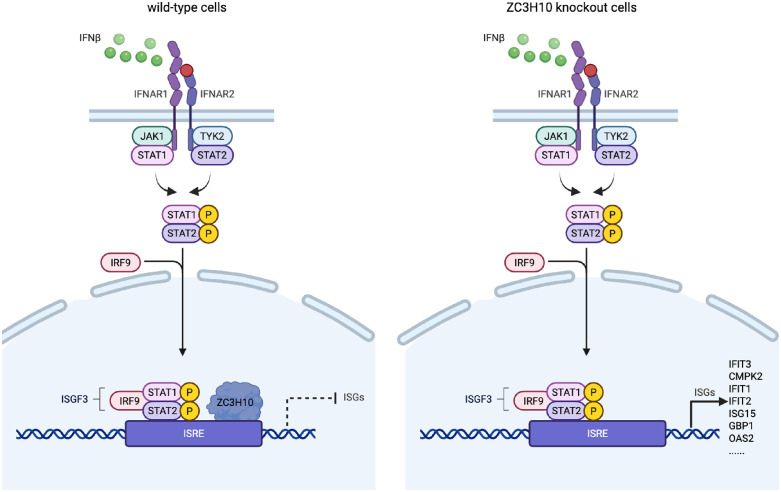
Schematic diagram illustrating the proposed mechanism by which ZC3H10 negatively regulates the interferon responses. In wild-type (WT) cells, ZC3H10 binds to the promoter region of ISRE-containing ISGs and inhibits their transcription. However, in ZC3H10 KO cells, the absence of ZC3H10-mediated inhibition leads to partial expression of ISGs even under basal conditions. Created in BioRender (https://BioRender.com/xzzya27).

In summary, we have identified ZC3H10 as a novel negative regulator of the type I IFN signaling pathway (Fig 7). ZC3H10 counteracts IFNβ’s antiviral effects by binding to TTTC sequences within ISG promoters, thereby suppressing ISG transcription in a manner dependent on its nuclear localization signal, zinc finger domains, coiled-coil domain, and the critical R89 residue. These insights deepen our understanding of the complex regulatory networks that control IFN signaling and highlight ZC3H10 as a potential target, one that could be inhibited to boost antiviral immunity or enhanced to manage autoimmune conditions characterized by IFN overproduction.

## Materials and methods

### Cell lines and viruses

A549 (CCL-185), HEK293A (CRL-1573), RAW264.7 (TIB-71), and 293T (CRL-3216) cells were purchased from the American Type Culture Collection (ATCC) and maintained in Dulbecco’s modified Eagle’s medium (Invitrogen, Carlsbad, CA), supplemented with 10% fetal bovine serum (HyClone, Logan, UT) and 100 U/ml penicillin and streptomycin at 37°C, 5% CO_2_. Newcastle disease virus-GFP (NDV-GFP) and human rhinovirus 16 (HRV16) were obtained from ATCC. Enterovirus A71 (EV71) is the Anhui Fuyang-0805 strain (GenBank accession no. FJ439769.1). The IPBCAMS-WH-01/2019 strain of SARS-CoV-2 was isolated and identified by our laboratory (GISAID: no. EPI_ISL_402123). Viral infection was carried out as described previously [48].

### Antibodies

Primary antibodies used were as follows: mouse anti-SARS-CoV/SARS-CoV-2 Nucleocapsid mAb from Sino Biological (Cat# 40143-MM08); mouse anti-Flag mAb from Sigma (Cat# F3165); mouse anti-β-actin mAb from Sigma (Cat# A5441); rabbit anti-ZC3H10 mAb from BGI Genomics Co., Ltd; rabbit anti-STAT1 mAb from Cell Signaling Technology (Cat#9172); mouse anti-Phospho-Stat1 (Tyr701) mAb from Thermo (Cat#QH219604); rabbit anti-STAT2 mAb from Cell Signaling Technology (Cat#72604); mouse anti-EV71 mAb from Millipore (Cat# 60062751); rabbit anti-GFP mAb from Sigma (Cat#G1544); mouse anti-HRV16 mAb from QED bioscience inc. (Cat#18758); mouse anti-IFIT2 mAb from Santa Cruz Biotechnology (Cat#Sc-390724).

### Plasmid

A cDNA library was purchased from OriGene Technologies Inc (plasmid backbone: pCMV6-xl4/xl5), as shown in S1 Table. The following plasmids were utilized and stored at the Christophe Merieux Laboratory: pCMV6-entry, pLenti-CRISPRv2, psPAX2, pVSVG. The ZC3H10 R89A mutant constructed using a Site-Directed Mutagenesis Kit (Stratagene, La Jolla, CA) according to the manufacturer’s instructions. The primers used for the R89A mutant were Forward: 5′- TGATCTCTATGATATCGCTGACCTTCCTGACAGGGGCTTTGA-3′, Reverse: 5′- CCCTGTCAGGAAGGTCAGCGATATCATAGAGATCATGACGTC-3′.

### Primary screening

HEK293A cells were seeded in 96-well plates. The following day, cells were transfected with cDNA plasmids using Lipofectamine 2000 (Invitrogen). At 24 hours post-transfection, the culture medium was replaced with Opti-MEM containing 400 U/mL of IFN-β. Cells were incubated with IFN-β for 12 hours. Then, the medium was removed, and cells were infected with NDV-GFP at an MOI of 1. After 16 hours, the supernatant was discarded, and 4% paraformaldehyde was added for fixation for 20 min. Following DAPI staining, fluorescence images were collected using the Operetta High Content Analysis System (PerkinElmer). The infection rate was calculated as: Infection rate = (GFP^+^ cell number/total cell number) × 100%. The Z-score was calculated as (infection rate − mean infection rate)/standard deviation.

### Reporter assays

293T cells were seeded in 24-well plates at a density of 2.5 × 10^5^ cells per well. The following day, cells were transfected using Lipofectamine 2000 (Invitrogen) with the following plasmids, 100 ng of pFL-ISRE-luciferase or pFL-IFIT1 promoter-luciferase, 5 ng of pRL-SV40, and 400 ng of the target gene plasmid. At 24 hours post-transfection, the culture medium was replaced with Opti-MEM containing 400 U/mL of IFN-β. Cell were incubated for 12 hours. Cells were harvested, and cell lysates were subjected to luciferase activity assays which were performed using the Dual-Luciferase Reporter Assay System (Promega). Activation degree was calculated as: Relative luciferase activity = Firefly fluorescence/Renilla fluorescence.

### SiRNA transfection for ZC3H10 knockdown

RAW 264.7 cells were seeded in 24-well plates at a density of 100,000 cells. After 24 hours, the cells were transfected with siRNA using the Lipofectamine RNAiMAX (Invitrogen), with a final siRNA concentration of 50 nM. Following 48 hours of incubation, the cells were harvested for subsequent RNA and protein expression analysis. The siRNA sequences used were as follows: GCCGUCAUGAUCUCUAUGATT and UCAUAGAGAUCAUGACGGCTT.

### Immunoblot analysis

Cells were lysed in RIPA buffer on ice for 30 min. Lysates were centrifuged at 12,000*g* for 10 min at 4 °C, and protein concentrations were determined using the BCA assay (Thermo Fisher Scientific, #23227). Proteins were denatured in loading buffer by boiling, separated by SDS‑PAGE, and transferred onto nitrocellulose membranes (Pall, #66485). Membranes were blocked for 1 h at room temperature with 5% non‑fat milk in TBST (Tris‑buffered saline with 0.1% Tween‑20), followed by incubation with primary antibodies overnight at 4 °C and then with appropriate IRDye‑conjugated secondary antibodies (Li-COR) for 30 min at room temperature. Signals were detected using an Odyssey infrared imaging system (Li‑COR) and analyzed with Odyssey Image Studio software. Source data are available in S1 Raw Images.

### Generation of HEK293A-ZC3H10 Knockout cell lines Using CRISPR-Cas9

The sequence of single-guide RNA (sgRNA) targeting ZC3H10 was 5′-ACAGCTATGCCAACGGTACC-3′. To package the ZC3H10-KO lentivirus, 293T cells were transfected with the following plasmids using Lipofectamine 2000: 1.2 μg of pLentiCRISPRv2-sgRNA, 0.9 μg of psPAX2, and 0.6 μg of pVSVG. After 72 h, lentivirus-containing supernatant was collected. HEK293A cells were infected with the packaged ZC3H10-KO lentivirus. After 12 hours, infected cells were cultured in DMEM, supplemented with 10% FBS and puromycin. After 48 hours, cells were separated into monoclonal populations and expanded to obtain cell lines. Genomic sequencing and western blot were performed to confirm ZC3H10 knockout efficiency.

### Real-time fluorescence quantitative PCR

Total RNA was extracted from cells using TRIzol reagent (Invitrogen) following the manufacturer’s instructions. Reverse transcription was performed using an MLV reverse transcription kit (Promega) according to the manufacturer’s protocol. The synthesized cDNA was used as a template for PCR amplification to detect the expression of target genes, including OAS1, OAS2, ISG15, IFIT2, and IFIT1. Primers used were as follows: human OAS1, TGTCCAAGGTGGTAAAGGGTG and CCGGCGATTTAACTGATCCTG; human OAS2, CTCAGAAGCTGGGTTGGTTTAT and ACCATCTCGTCGATCAGTGTC; human ISG15, CGCAGATCACCCAGAAGATCG and TTCGTCGCATTTGTCCACCA; human IFIT2, CTGCAACCATGAGTGAGAA and CCTTTGAGGTGCTTTAGATAG; human IFIT1, TACAGCAACCATGAGTACAA and TCAGGTGTTTCACATAGGC; human GAPDH, CGGAGTCAACGGATTTGGTCGTA and AGCCTTCTCCATGGTGGTGAAGAC.

### RNA sequencing and data analysis

Total RNA was extracted from three biological replicates each of wild-type and ZC3H10-knockout cells using TRIzol reagent (Invitrogen, USA). One microgram of total RNA from each sample was used as input for RNA-seq library preparation. Libraries were constructed using the Hieff NGS MaxUp mRNA Library Prep Kit for Illumina (Yeasen, China). Briefly, mRNA was enriched from total RNA using poly-T oligo-attached magnetic beads, fragmented, and reverse transcribed into first-strand cDNA, followed by second-strand cDNA synthesis. After end repair, adaptor ligation, USER enzyme treatment, and PCR amplification, the libraries were purified and quality assessed using an Agilent 2100 Bioanalyzer (Agilent Technologies, USA). Sequencing was performed on an Illumina HiSeq 2500 platform (Illumina, UK).

Raw reads were processed with Fastp for adapter trimming and quality filtering. Clean reads were aligned to the human reference genome GRCh38 using HISAT2, and gene-level counts were generated with featureCounts. Low-expression genes (average CPM < 5 in both groups and detected in < 50% of samples) were filtered out. Differential expression analysis was conducted in R using Bioconductor DESeq2 package (FDR < 0.05, |log₂FC| > 1). Results were visualized with a volcano plot (Bioconductor ggplot2 package. GO enrichment analysis for up-regulated genes was performed using the Bioconductor clusterProfiler package, and results were displayed as bubble charts (ggplot2). A heatmap of upregulated interferon-stimulated genes (ISGs) was generated with Bioconductor heatmap package.

### DNA pull-down assay

293T cells were transfected with ZC3H10-Flag plasmids. After 16 hours, cells were scraped off, and nucleoproteins were extracted. Double-stranded DNA (dsDNA) was prepared by annealing non-biotinylated or biotinylated single-stranded DNA (ssDNA). The annealing process was done by heating the ssDNA at 95 °C for 10 min, followed by slow cooling to room temperature. Ten µg dsDNA or ssDNA was mixed with 75 µL of MyOne C1 in 400 µL DB buffer (20mM Tris-HCl (pH8.0), 2M NaCl, 0.5mM EDTA, 0.03% NP40). The mixture was incubated at room temperature for 1 hour to allow DNA binding to the beads. The DNA-linked beads were washed with 1 mL of DB buffer and 1 mL of PB buffer (50 mM Tris-HCl (pH 8.0), 150 mM NaCl, 10 mM MgCl_2_, 0.5% NP-40, and a proteinase inhibitor mixture). The washed DNA-linked beads were incubated with 400 µg of nucleoprotein in PB buffer for 2 hours at room temperature, resulting in a total volume of 600 µL. The beads were washed three times with 1 mL of PB buffer. Then, the beads were eluted with 25 μL of 0.1% SDS solution and subjected to immunoblotting.

### Immunofluorescence

Cells were fixed with 4% paraformaldehyde at room temperature for 20 min. After fixation, the cells were washed with 1 mL of PBS. 1 mL of 0.5% Triton X-100 (prepared with PBS) was added to the cells and incubated at room temperature for 10 min. After washing three times with PBS, 1 mL of 5% BSA (prepared with PBST) was added and incubated at room temperature for 90 min to block non-specific binding. The primary antibody was diluted with 5% BSA and incubated with the cells overnight at 4 °C. The cells were washed three times with PBST and incubated with the diluted secondary antibody for 1 hour at room temperature. Lastly, the cells were washed three times with PBST and incubated with DAPI at room temperature for 10 min.

### Chromatin Immunoprecipitation Sequencing (ChIP-Seq) assay

A549 cells were seeded in 10 cm dishes at a density of 5 × 10⁶ cells per dish. The following day, cells were transfected with 10 µg of either empty vectors or vectors expressing Flag-tagged ZC3H10. ChIP-seq experiments were performed by Biomarker Technologies Co. (Beijing, China) according to a standard crosslinking chromatin immunoprecipitation protocol with minor modifications. Cells were crosslinked with 1% formaldehyde for 10 min at room temperature. Chromatin was isolated and fragmented by sonication. Immunoprecipitation was performed using anti-Flag gels to enrich ZC3H10-associated chromatin fragments. Following extensive washing, DNA-protein complexes were eluted, and DNA was purified using the MinElute PCR Purification Kit (QIAGEN, #28006). ChIP-seq libraries were prepared using the TruePrep DNA Library Prep Kit V2 for Illumina (Vazyme, #TD501) according to the manufacturer’s instructions. Purified DNA fragments were ligated to sequencing adaptors and amplified for library construction. Libraries were subjected to paired-end 150-bp sequencing on an Illumina NovaSeq 6000 platform.

Raw sequencing reads were processed for adapter trimming and quality filtering. Clean reads were aligned to the human reference genome (GRCh38), and enriched binding regions (peaks) were identified using standard ChIP-seq analysis pipelines. Differential binding analysis was performed in R using the Bioconductor DiffBind package to identify peaks with significant changes in enrichment between experimental conditions.

### Statistics

Statistical significance was determined using Student *t* test or one-way ANOVA, calculated using GraphPad Prism software. Data are presented as mean ± SD. Statistical significance was determined as *P < 0.05, **P < 0.01, ***P < 0.001, or ns, *P* > 0.05.

## Supporting information

S1 Fig ZC3H10 selectively suppresses IFN signaling without affecting non-ISG expression or inflammatory responses. (TIF)

S2 Fig ZC3H10-mediated repression of ISGs is independent of IFNAR expression and mRNA stability. (TIF)

S3 Fig ZC3H10 preferentially recognizes ISRE-containing promoter sequences, and Ser126 is dispensable for its repressive activity. (TIF)

S1 Table Z-score values for all genes identified in the primary cDNA library screen. (XLSX)

S2 Table Transcriptomic analysis of wild-type and ZC3H10-knockout HEK293A cells. (XLSX)

S3 Table GO enrichment analysis of genes upregulated in ZC3H10-knockout cells. (XLSX)

S1 Data Raw numerical data underlying Figs 1–6 and S1–S3. Each worksheet contains the source data for the corresponding figure panels, including all individual replicate values used for statistical analyses.
(XLSX)

S1 Raw Images Unedited original Western blots and gels used in the study. (PDF)

## Abbreviations

ATCC: American Type Culture Collection
cDNA: complementary DNA
ChIP-Seq: chromatin immunoprecipitation sequencing
cGAS: cyclic GMP-AMP synthase
DE-ISGs: differentially expressed ISGs
dsDNA: double-stranded DNA
EHMT2: euchromatic histone lysine methyltransferase 2
GFP: green fluorescence protein
GO: Gene Ontology
HDACs: histone deacetylases
IFN: interferon
IFNAR: interferon alpha/beta receptor
IRF: interferon regulatory factor
ISGs: interferon-stimulated genes
ISGF3: interferon-stimulated gene factor 3
ISREs: interferon-stimulated response elements
JAK1: Janus kinase 1
KO: knockout
MDA5: melanoma differentiation-associated protein 5
NDV: Newcastle disease virus
NF-κB: nuclear factor kappa B
NLS: nuclear localization signal
PAMPs: pathogen-associated molecular patterns
PGM2: phosphoglucomutase 2
PRRs: pattern recognition receptors
qRT-PCR: quantitative real-time PCR
RIG-I: retinoic acid-inducible gene I
RLRs: retinoic acid-inducible gene I-like receptors
RNA-Seq: RNA sequencing
RPA: replication protein A
SARS-CoV-2: severe acute respiratory syndrome coronavirus 2
sgRNA: single-guide RNA
SLE: systemic lupus erythematosus
SOCS: suppressor of cytokine signaling
ssDNA: single-stranded DNA
STAT1: signal transducer and activator of transcription 1
TLRs: Toll-like receptors
TYK2: tyrosine kinase 2
USP18: ubiquitin carboxy-terminal hydrolase 18
WT: wild-type

## References

[1] Akira S, Uematsu S, Takeuchi O. Pathogen recognition and innate immunity. Cell. 2006;124(4):783–801. doi: 10.1016/j.cell.2006.02.015 16497588

[2] Pradeu T, Thomma BPHJ, Girardin SE, Lemaitre B. The conceptual foundations of innate immunity: taking stock 30 years later. Immunity. 2024;57(4):613–31. doi: 10.1016/j.immuni.2024.03.007 38599162

[3] Kato H, Sato S, Yoneyama M, Yamamoto M, Uematsu S, Matsui K, et al. Cell type-specific involvement of RIG-I in antiviral response. Immunity. 2005;23(1):19–28. doi: 10.1016/j.immuni.2005.04.010 16039576

[4] Yoneyama M, Kikuchi M, Natsukawa T, Shinobu N, Imaizumi T, Miyagishi M, et al. The RNA helicase RIG-I has an essential function in double-stranded RNA-induced innate antiviral responses. Nat Immunol. 2004;5(7):730–7. doi: 10.1038/ni1087 15208624

[5] Fitzgerald KA, Kagan JC. Toll-like receptors and the control of immunity. Cell. 2020;180(6):1044–66. doi: 10.1016/j.cell.2020.02.041 32164908 PMC9358771

[6] Kawai T, Ikegawa M, Ori D, Akira S. Decoding Toll-like receptors: Recent insights and perspectives in innate immunity. Immunity. 2024;57(4):649–73. doi: 10.1016/j.immuni.2024.03.004 38599164

[7] Girardin SE, Boneca IG, Carneiro LAM, Antignac A, Jéhanno M, Viala J, et al. Nod1 detects a unique muropeptide from gram-negative bacterial peptidoglycan. Science. 2003;300(5625):1584–7. doi: 10.1126/science.1084677 12791997

[8] Kanneganti T-D, Ozören N, Body-Malapel M, Amer A, Park J-H, Franchi L, et al. Bacterial RNA and small antiviral compounds activate caspase-1 through cryopyrin/Nalp3. Nature. 2006;440(7081):233–6. doi: 10.1038/nature04517 16407888

[9] Chen Q, Sun L, Chen ZJ. Regulation and function of the cGAS-STING pathway of cytosolic DNA sensing. Nat Immunol. 2016;17(10):1142–9. doi: 10.1038/ni.3558 27648547

[10] Sun L, Wu J, Du F, Chen X, Chen ZJ. Cyclic GMP-AMP synthase is a cytosolic DNA sensor that activates the type I interferon pathway. Science. 2013;339(6121):786–91. doi: 10.1126/science.1232458 23258413 PMC3863629

[11] Gao D, Wu J, Wu Y-T, Du F, Aroh C, Yan N, et al. Cyclic GMP-AMP synthase is an innate immune sensor of HIV and other retroviruses. Science. 2013;341(6148):903–6. doi: 10.1126/science.1240933 23929945 PMC3860819

[12] Ivashkiv LB, Donlin LT. Regulation of type I interferon responses. Nat Rev Immunol. 2014;14(1):36–49. doi: 10.1038/nri3581 24362405 PMC4084561

[13] Li D, Wu M. Pattern recognition receptors in health and diseases. Signal Transduct Target Ther. 2021;6(1):291. doi: 10.1038/s41392-021-00687-0 34344870 PMC8333067

[14] Philips RL, Wang Y, Cheon H, Kanno Y, Gadina M, Sartorelli V, et al. The JAK-STAT pathway at 30: Much learned, much more to do. Cell. 2022;185(21):3857–76. doi: 10.1016/j.cell.2022.09.023 36240739 PMC9815833

[15] Levy DE, Darnell JE Jr. Stats: transcriptional control and biological impact. Nat Rev Mol Cell Biol. 2002;3(9):651–62. doi: 10.1038/nrm909 12209125

[16] Schneider WM, Chevillotte MD, Rice CM. Interferon-stimulated genes: a complex web of host defenses. Annu Rev Immunol. 2014;32:513–45. doi: 10.1146/annurev-immunol-032713-120231 24555472 PMC4313732

[17] MacMicking JD. Interferon-inducible effector mechanisms in cell-autonomous immunity. Nat Rev Immunol. 2012;12(5):367–82. doi: 10.1038/nri3210 22531325 PMC4150610

[18] Fernbach S, Spieler EE, Busnadiego I, Karakus U, Lkharrazi A, Stertz S, et al. Restriction factor screening identifies RABGAP1L-mediated disruption of endocytosis as a host antiviral defense. Cell Rep. 2022;38(12):110549. doi: 10.1016/j.celrep.2022.110549 35320721 PMC8939003

[19] Kuroda M, Halfmann PJ, Hill-Batorski L, Ozawa M, Lopes TJS, Neumann G, et al. Identification of interferon-stimulated genes that attenuate Ebola virus infection. Nat Commun. 2020;11(1):2953. doi: 10.1038/s41467-020-16768-7 32528005 PMC7289892

[20] Ma R, Zhang X, Li R, Dong X, Wang W, Jiang Q, et al. PLSCR1 suppresses SARS-CoV-2 infection by downregulating cell surface ACE2. J Virol. 2025;99(3):e0208524. doi: 10.1128/jvi.02085-24 39945535 PMC11915802

[21] Mostafavi S, Yoshida H, Moodley D, LeBoité H, Rothamel K, Raj T, et al. Parsing the interferon transcriptional network and its disease associations. Cell. 2016;164(3):564–78. doi: 10.1016/j.cell.2015.12.032 26824662 PMC4743492

[22] Crow YJ, Casanova J-L. Human life within a narrow range: the lethal ups and downs of type I interferons. Sci Immunol. 2024;9(97):eadm8185. doi: 10.1126/sciimmunol.adm8185 38968338

[23] Crow MK, Olferiev M, Kirou KA. Type I interferons in autoimmune disease. Annu Rev Pathol. 2019;14:369–93. doi: 10.1146/annurev-pathol-020117-043952 30332560

[24] Crow MK. Pathogenesis of systemic lupus erythematosus: risks, mechanisms and therapeutic targets. Ann Rheum Dis. 2023;82(8):999–1014. doi: 10.1136/ard-2022-223741 36792346

[25] Lin CMA, Isaacs JD, Cooles FAH. Role of IFN-α in rheumatoid arthritis. Curr Rheumatol Rep. 2024;26(2):37–52. doi: 10.1007/s11926-023-01125-6 38051494 PMC10787895

[26] Kakkar V, Assassi S, Allanore Y, Kuwana M, Denton CP, Khanna D, et al. Type 1 interferon activation in systemic sclerosis: a biomarker, a target or the culprit. Curr Opin Rheumatol. 2022;34(6):357–64. doi: 10.1097/BOR.0000000000000907 36125916 PMC9594133

[27] Xia C, Wolf JJ, Sun C, Xu M, Studstill CJ, Chen J, et al. PARP1 enhances influenza A virus propagation by facilitating degradation of host type I interferon receptor. J Virol. 2020;94(7):e01572-19. doi: 10.1128/JVI.01572-19 31915279 PMC7081902

[28] Xia C, Vijayan M, Pritzl CJ, Fuchs SY, McDermott AB, Hahm B. Hemagglutinin of influenza A virus antagonizes type I interferon (IFN) responses by inducing degradation of type I IFN receptor 1. J Virol. 2015;90(5):2403–17. doi: 10.1128/JVI.02749-15 26676772 PMC4810695

[29] Hong XX, Carmichael GG. Innate immunity in pluripotent human cells: attenuated response to interferon-β. J Biol Chem. 2013;288:16196–205. 23599426 10.1074/jbc.M112.435461 PMC3668775

[30] Zhang JG, Metcalf D, Rakar S, Asimakis M, Greenhalgh CJ, Willson TA, et al. The SOCS box of suppressor of cytokine signaling-1 is important for inhibition of cytokine action in vivo. Proc Natl Acad Sci U S A. 2001;98(23):13261–5. doi: 10.1073/pnas.231486498 11606785 PMC60858

[31] Kershaw NJ, Murphy JM, Liau NPD, Varghese LN, Laktyushin A, Whitlock EL, et al. SOCS3 binds specific receptor-JAK complexes to control cytokine signaling by direct kinase inhibition. Nat Struct Mol Biol. 2013;20(4):469–76. doi: 10.1038/nsmb.2519 23454976 PMC3618588

[32] Martin-Fernandez M, Buta S, Le Voyer T, Li Z, Dynesen LT, Vuillier F, et al. A partial form of inherited human USP18 deficiency underlies infection and inflammation. J Exp Med. 2022;219(4):e20211273. doi: 10.1084/jem.20211273 35258551 PMC8908790

[33] Malakhova OA, Kim KI, Luo J-K, Zou W, Kumar KGS, Fuchs SY, et al. UBP43 is a novel regulator of interferon signaling independent of its ISG15 isopeptidase activity. EMBO J. 2006;25(11):2358–67. doi: 10.1038/sj.emboj.7601149 16710296 PMC1478183

[34] Nazarov PV, Reinsbach SE, Muller A, Nicot N, Philippidou D, Vallar L, et al. Interplay of microRNAs, transcription factors and target genes: linking dynamic expression changes to function. Nucleic Acids Res. 2013;41(5):2817–31. doi: 10.1093/nar/gks1471 23335783 PMC3597666

[35] Lian H, Zang R, Wei J, Ye W, Hu M-M, Chen Y-D, et al. The zinc-finger protein ZCCHC3 binds RNA and facilitates viral RNA sensing and activation of the RIG-I-like receptors. Immunity. 2018;49(3):438-448.e5. doi: 10.1016/j.immuni.2018.08.014 30193849

[36] Fu M, Blackshear PJ. RNA-binding proteins in immune regulation: a focus on CCCH zinc finger proteins. Nat Rev Immunol. 2017;17(2):130–43. doi: 10.1038/nri.2016.129 27990022 PMC5556700

[37] Audano M, Pedretti S, Cermenati G, Brioschi E, Diaferia GR, Ghisletti S, et al. Zc3h10 is a novel mitochondrial regulator. EMBO Rep. 2018;19(4):e45531. doi: 10.15252/embr.201745531 29507079 PMC5891430

[38] Treiber T, Treiber N, Plessmann U, Harlander S, Daiß J-L, Eichner N, et al. A compendium of RNA-binding proteins that regulate microRNA biogenesis. Mol Cell. 2017;66(2):270-284.e13. doi: 10.1016/j.molcel.2017.03.014 28431233

[39] Yi D, Dempersmier JM, Nguyen HP, Viscarra JA, Dinh J, Tabuchi C, et al. Zc3h10 acts as a transcription factor and is phosphorylated to activate the thermogenic program. Cell Rep. 2019;29(9):2621-2633.e4. doi: 10.1016/j.celrep.2019.10.099 31775033 PMC6911170

[40] Hanada T, Yoshida H, Kato S, Tanaka K, Masutani K, Tsukada J, et al. Suppressor of cytokine signaling-1 is essential for suppressing dendritic cell activation and systemic autoimmunity. Immunity. 2003;19(3):437–50. doi: 10.1016/s1074-7613(03)00240-1 14499118

[41] Hadjadj J, Castro CN, Tusseau M, Stolzenberg M-C, Mazerolles F, Aladjidi N, et al. Early-onset autoimmunity associated with SOCS1 haploinsufficiency. Nat Commun. 2020;11(1):5341. doi: 10.1038/s41467-020-18925-4 33087723 PMC7578789

[42] Arimoto K-I, Löchte S, Stoner SA, Burkart C, Zhang Y, Miyauchi S, et al. STAT2 is an essential adaptor in USP18-mediated suppression of type I interferon signaling. Nat Struct Mol Biol. 2017;24(3):279–89. doi: 10.1038/nsmb.3378 28165510 PMC5365074

[43] Zhang X, Cheng L, Gao C, Chen J, Liao S, Zheng Y, et al. Androgen signaling contributes to sex differences in cancer by inhibiting NF-κB activation in T cells and suppressing antitumor immunity. Cancer Res. 2023;83(6):906–21. doi: 10.1158/0008-5472.CAN-22-2405 36634207

[44] Liu X, Li H, Zhong B, Blonska M, Gorjestani S, Yan M, et al. USP18 inhibits NF-κB and NFAT activation during Th17 differentiation by deubiquitinating the TAK1-TAB1 complex. J Exp Med. 2013;210(8):1575–90. doi: 10.1084/jem.20122327 23825189 PMC3727316

[45] Yang Z, Xian H, Hu J, Tian S, Qin Y, Wang R-F, et al. USP18 negatively regulates NF-κB signaling by targeting TAK1 and NEMO for deubiquitination through distinct mechanisms. Sci Rep. 2015;5:12738. doi: 10.1038/srep12738 26240016 PMC4523862

[46] Taura M, Frank JA, Takahashi T, Kong Y, Kudo E, Song E, et al. APOBEC3A regulates transcription from interferon-stimulated response elements. Proc Natl Acad Sci U S A. 2022;119(20):e2011665119. doi: 10.1073/pnas.2011665119 35549556 PMC9171812

[47] Wold MS. Replication protein A: a heterotrimeric, single-stranded DNA-binding protein required for eukaryotic DNA metabolism. Annu Rev Biochem. 1997;66:61–92. doi: 10.1146/annurev.biochem.66.1.61 9242902

[48] Lei X, Dong X, Ma R, Wang W, Xiao X, Tian Z, et al. Activation and evasion of type I interferon responses by SARS-CoV-2. Nat Commun. 2020;11(1):3810. doi: 10.1038/s41467-020-17665-9 32733001 PMC7392898

