# Supplementary figures and images for: The zinc-finger protein ZC3H10 suppresses type I interferon responses during viral infection by repressing interferon-stimulated gene promoters
Source: PLoS Biol. 2026 Jul 16;24(7):e3003881. doi: 10.1371/journal.pbio.3003881 (PMC13375009; doi:10.1371/journal.pbio.3003881)

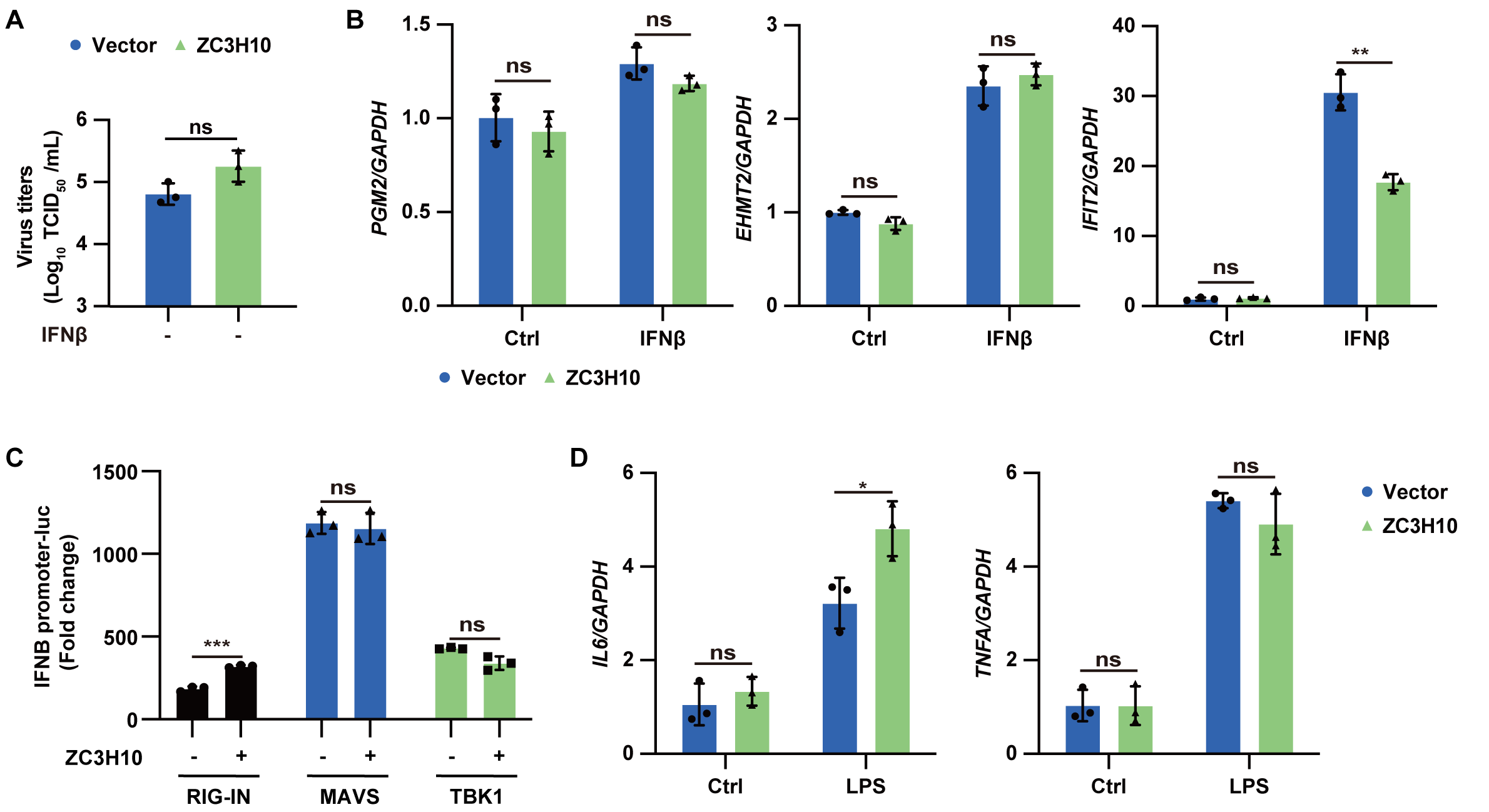

Supplement: S1 Fig — (TIF) [file pbio.3003881.s001.tif]

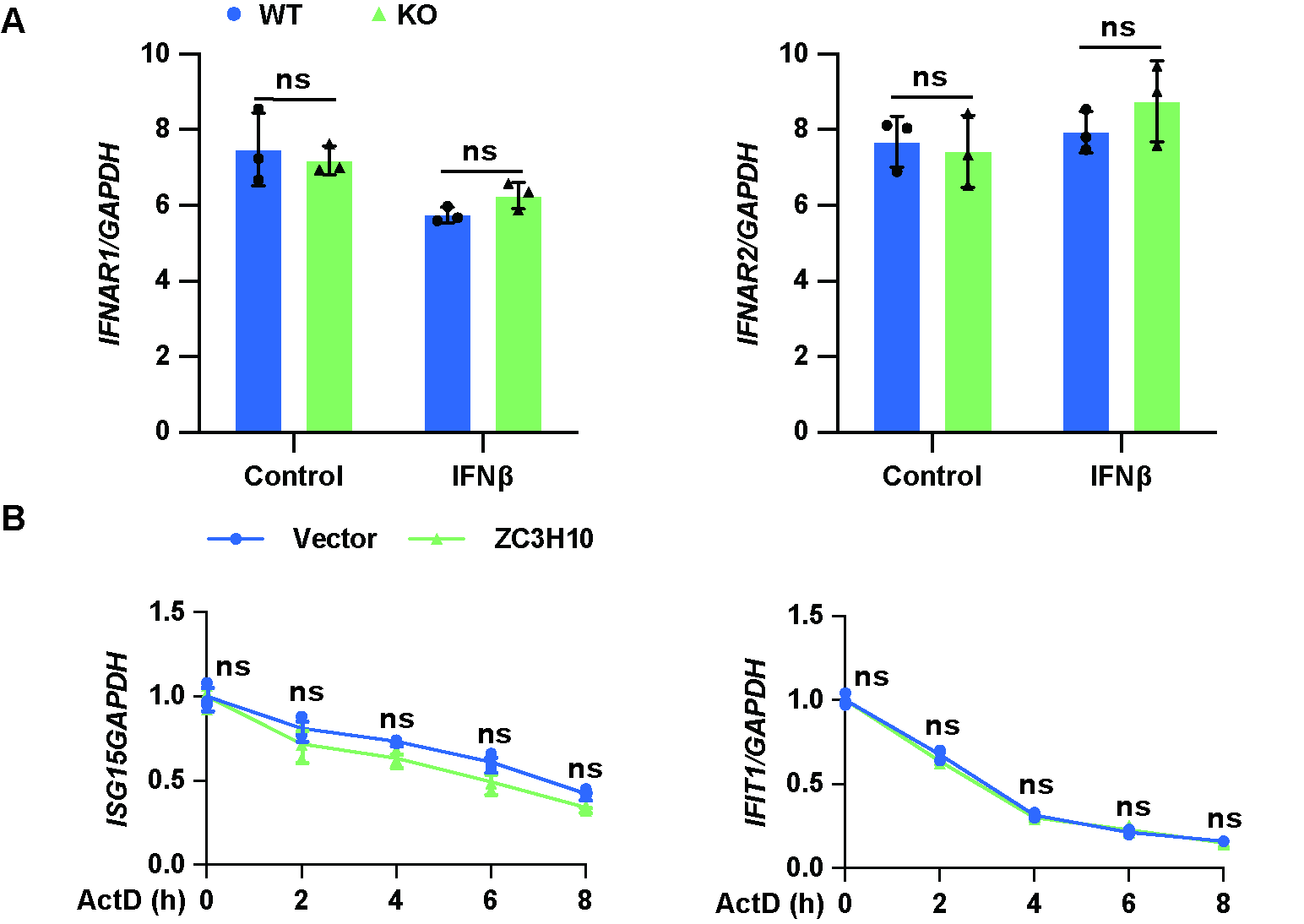

Supplement: S2 Fig — (TIF) [file pbio.3003881.s002.tif]

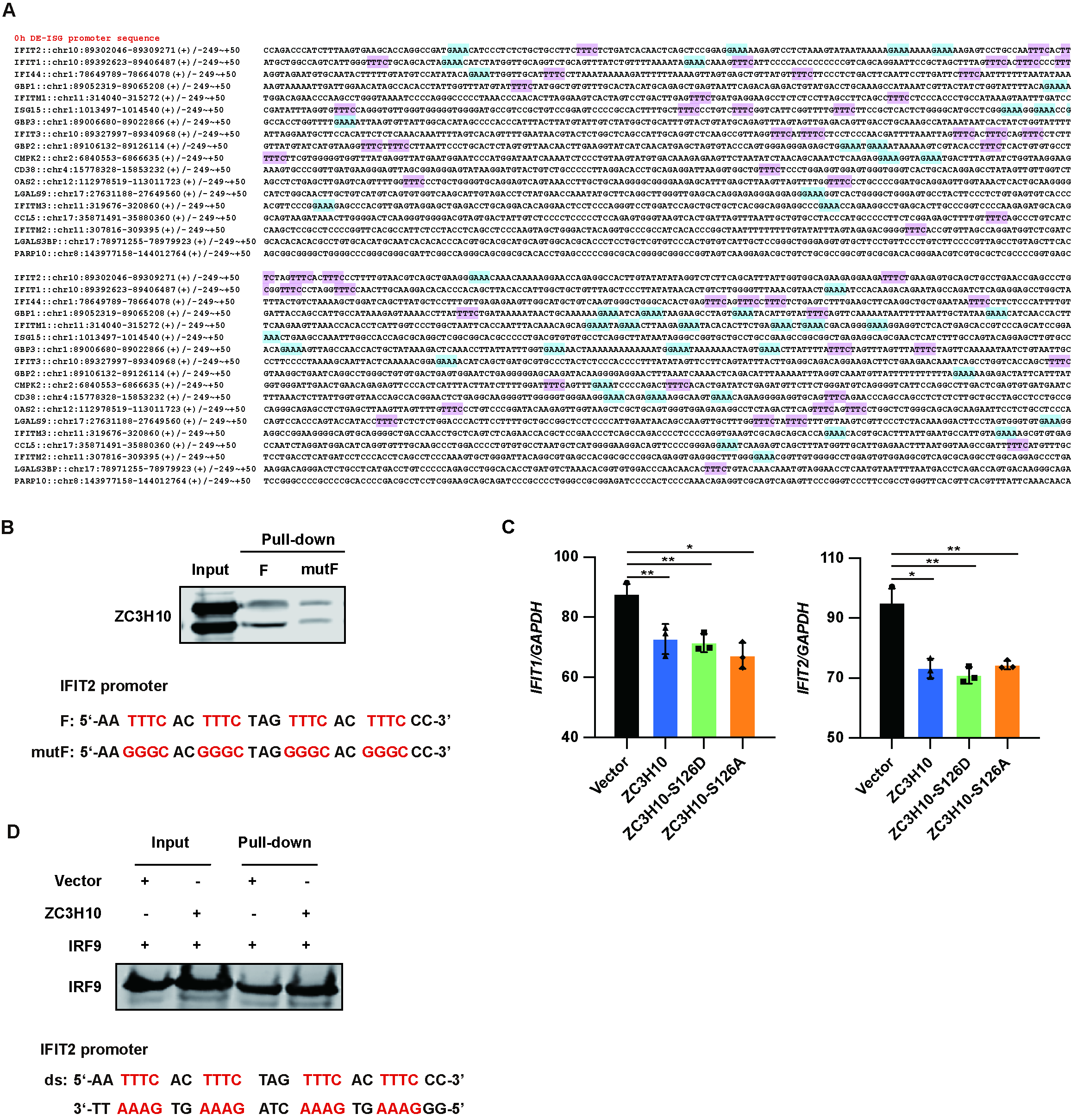

Supplement: S3 Fig — (TIF) [file pbio.3003881.s003.tif]

Fig. 2

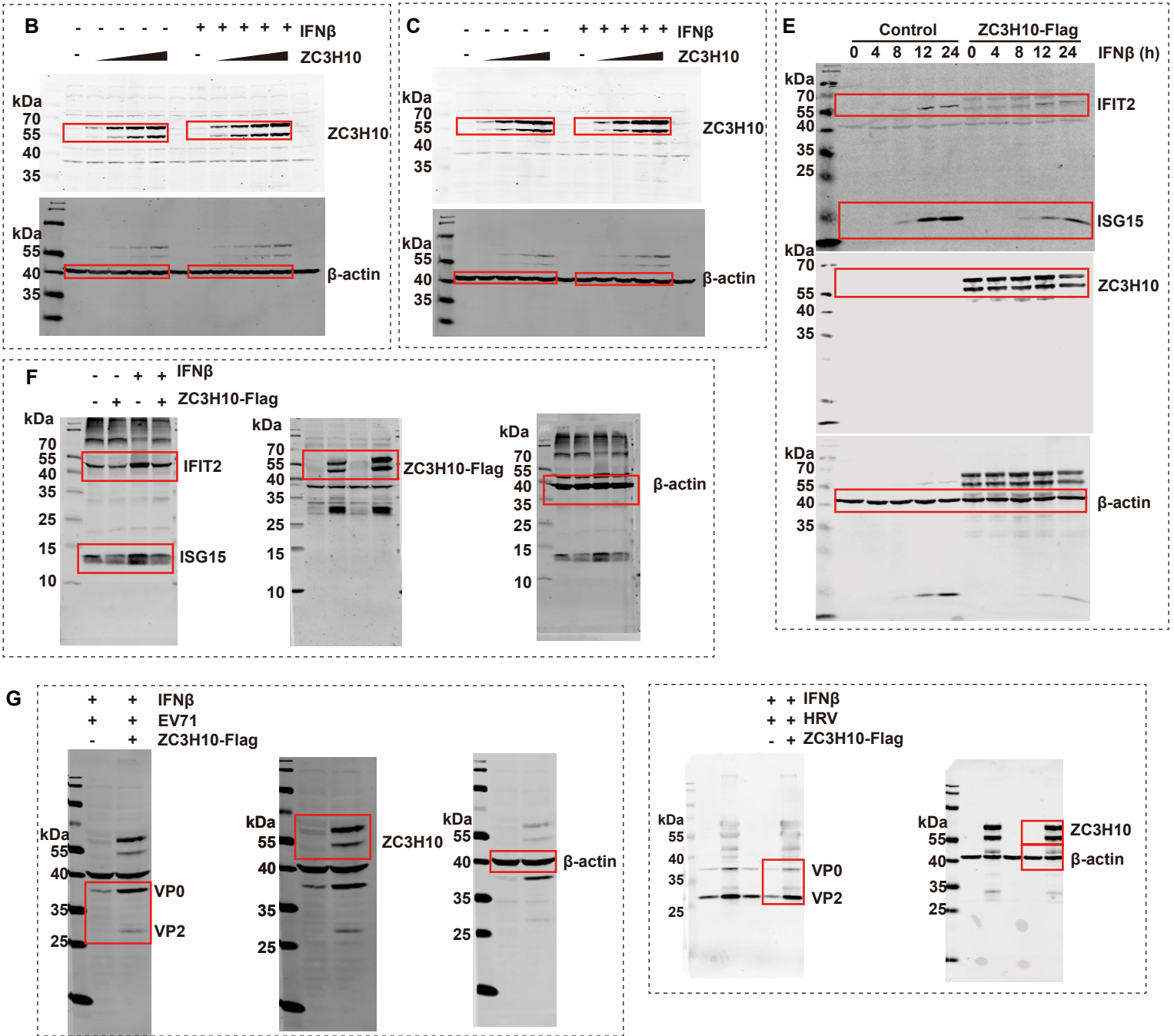

Fig. 3

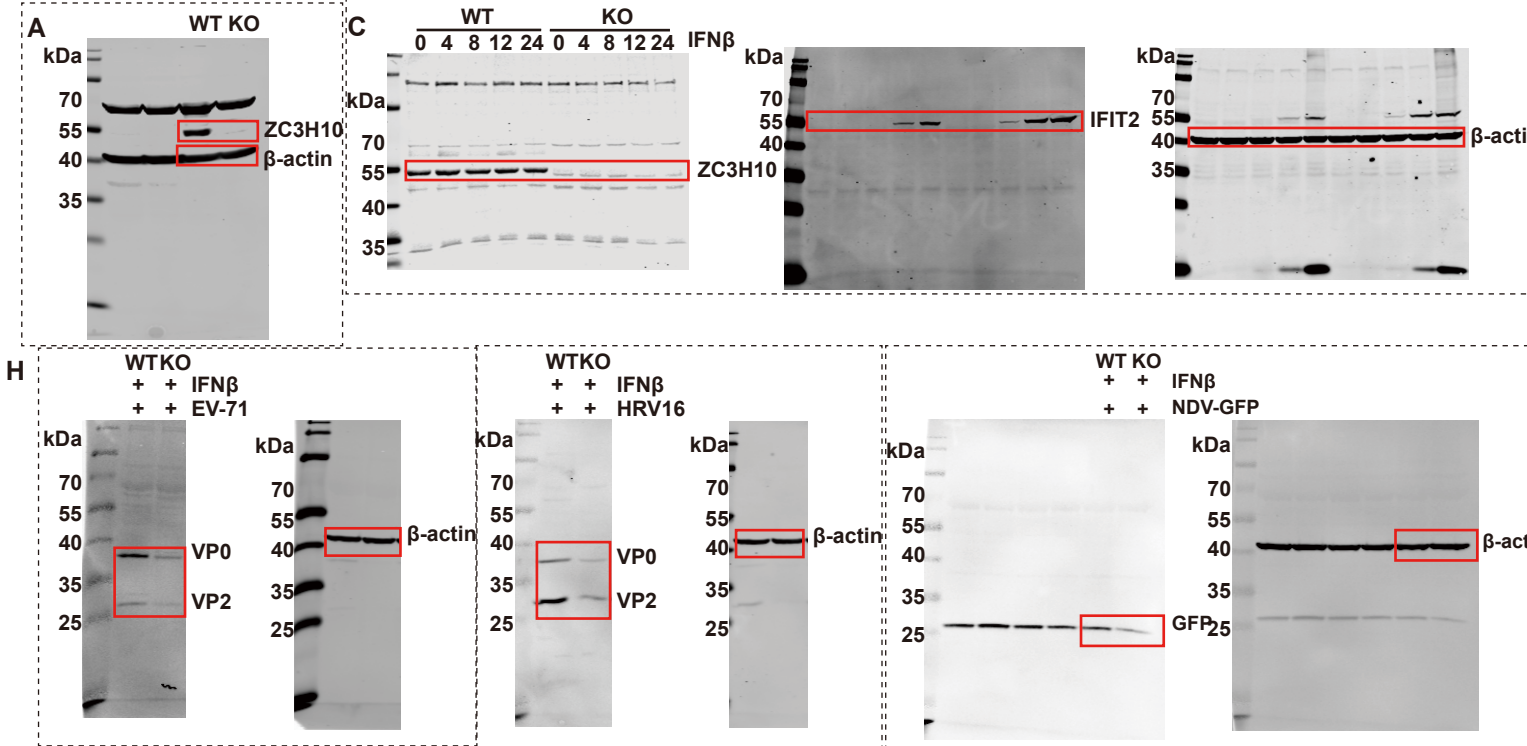

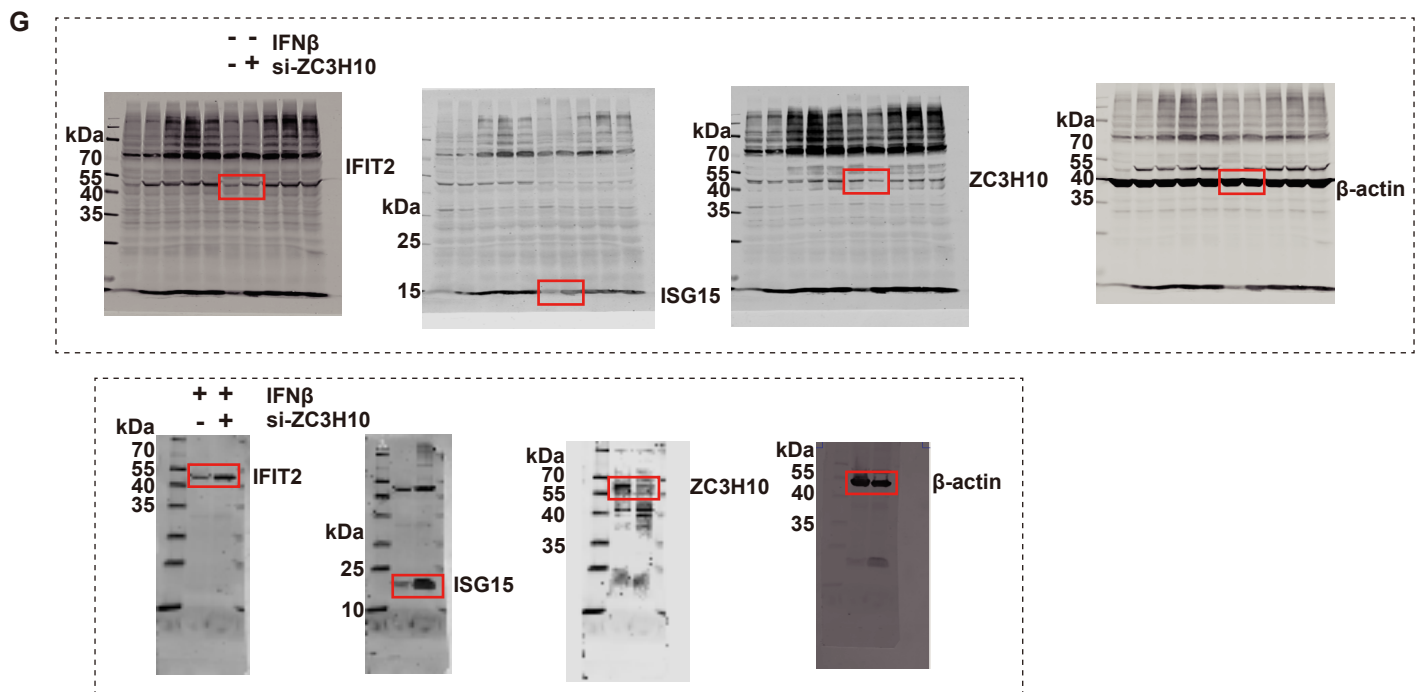

Fig. 4

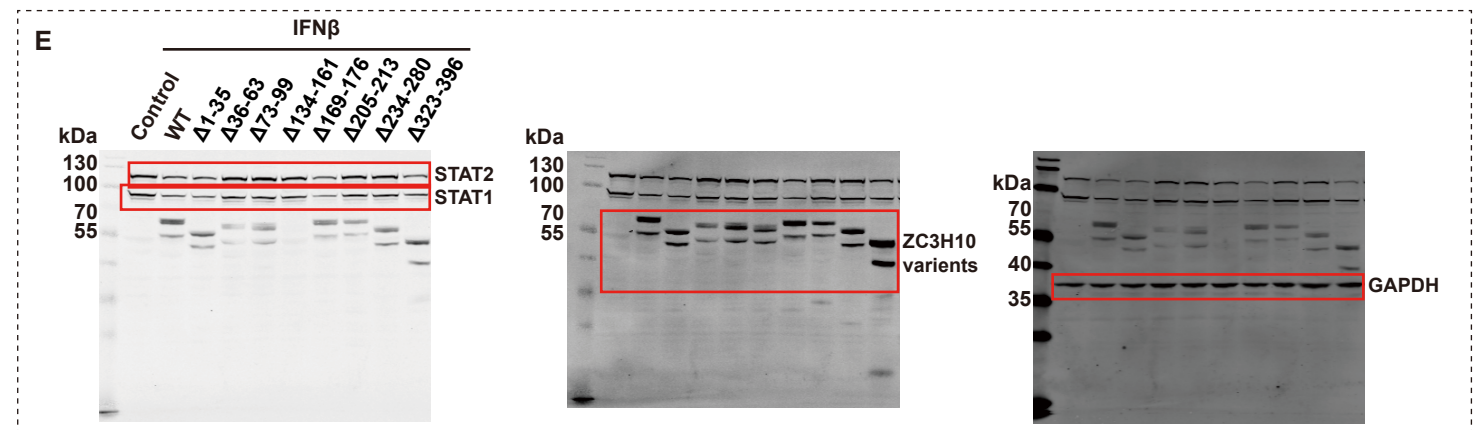

Fig. 5

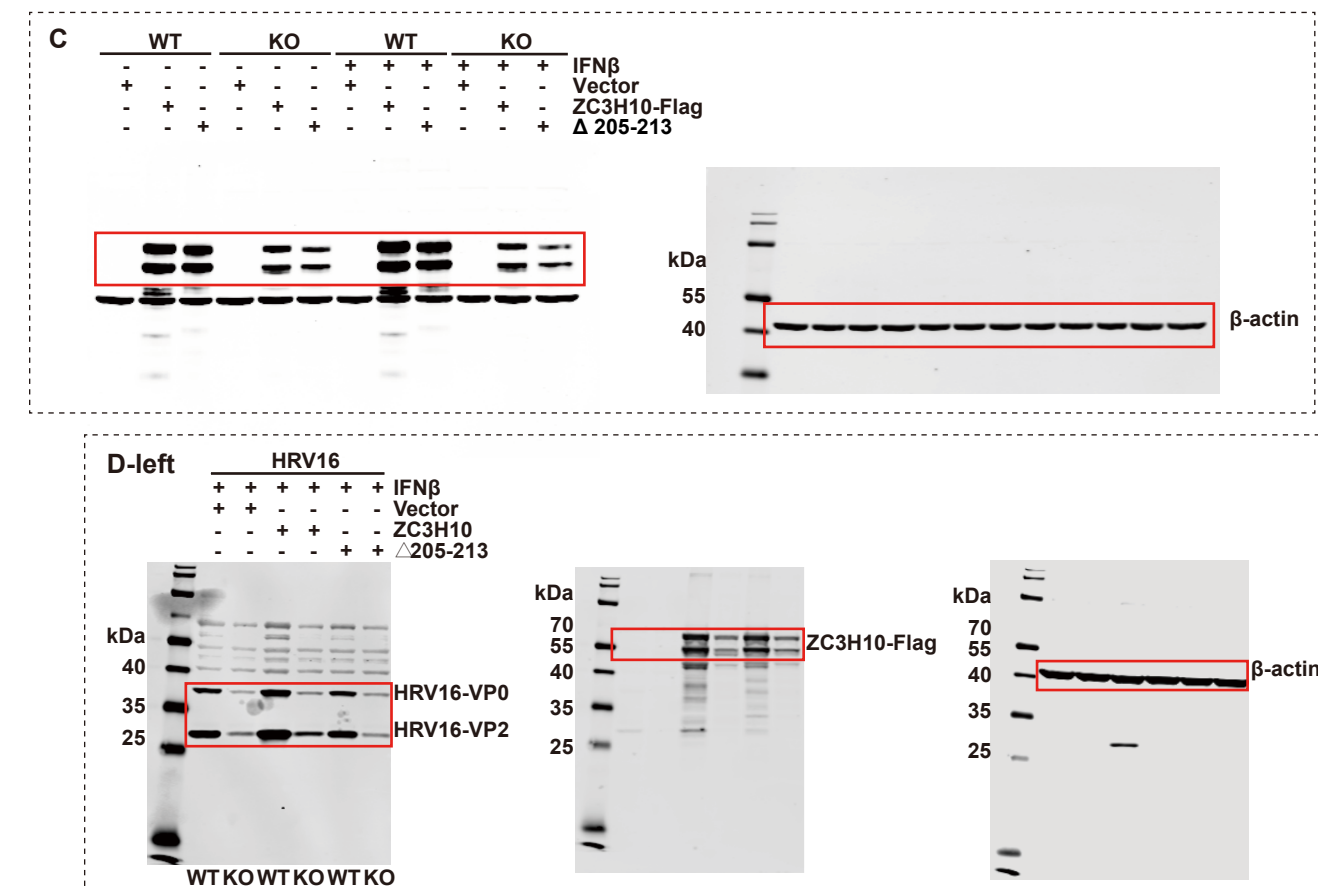

D-right

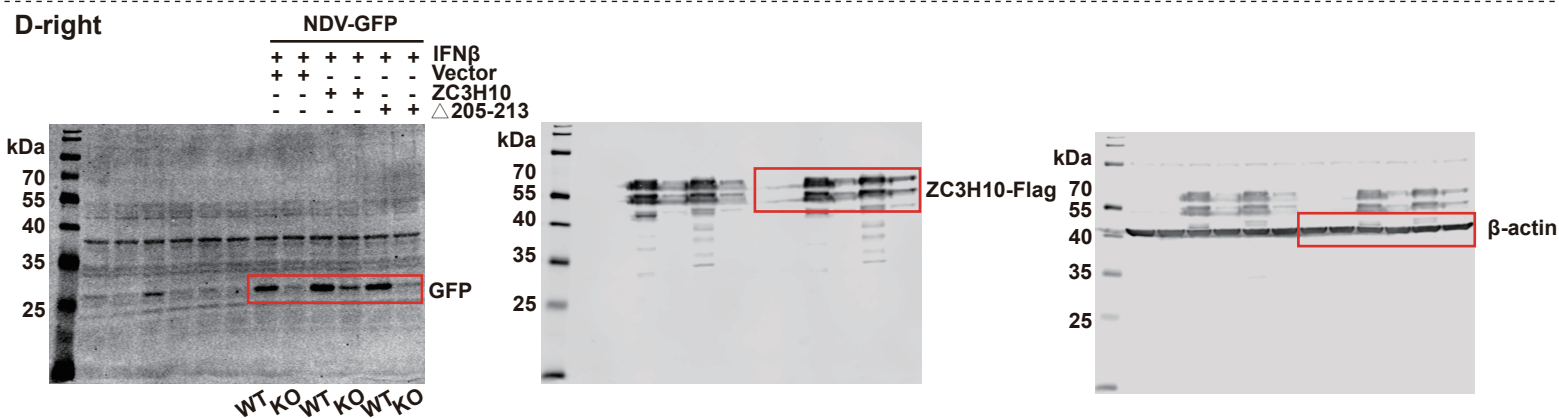

Fig. 6

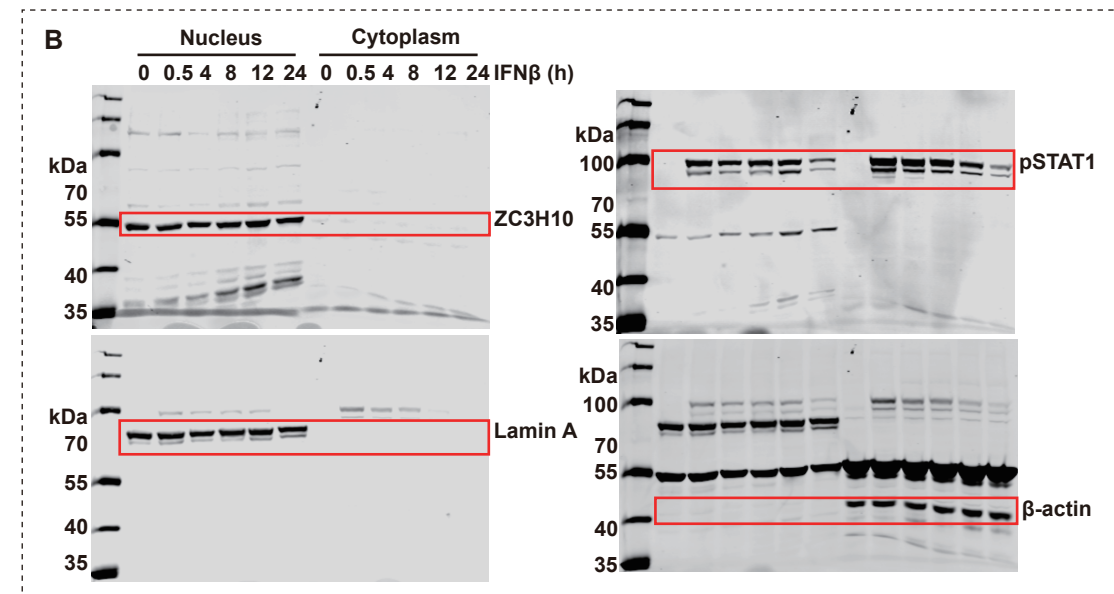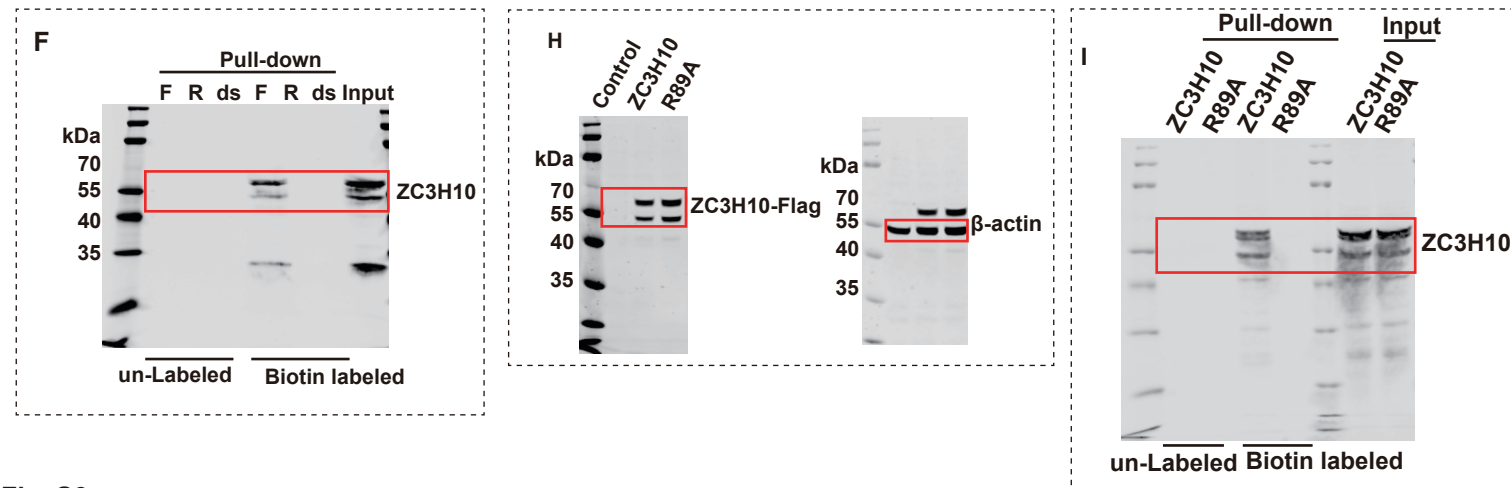

Fig. S3

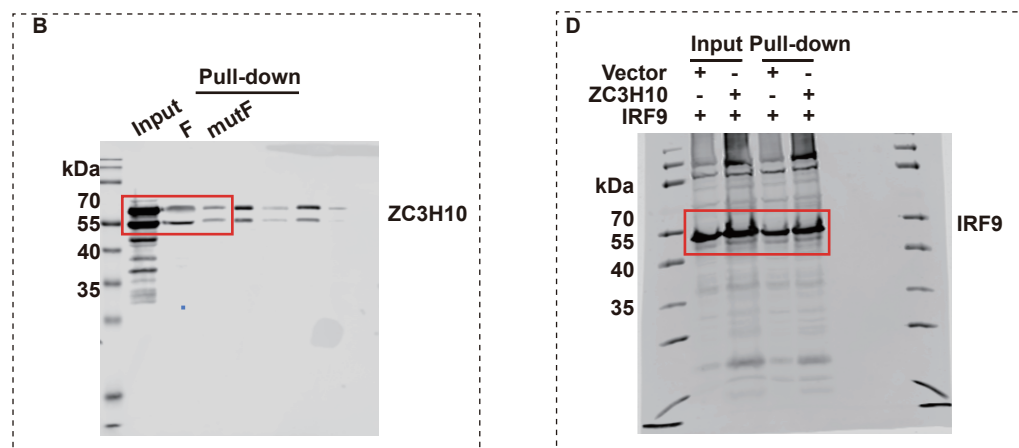

Supplement: S1 Raw Images — (PDF) [file pbio.3003881.s008.pdf]
